# A Mixed Scoping and Narrative Review of Immersive Technologies Applied to Patients for Pain, Anxiety, and Distress in Radiology and Radiotherapy

**DOI:** 10.3390/diagnostics15172174

**Published:** 2025-08-27

**Authors:** Andrea Lastrucci, Nicola Iosca, Giorgio Busto, Yannick Wandael, Angelo Barra, Mirko Rossi, Ilaria Morelli, Antonia Pirrera, Isacco Desideri, Renzo Ricci, Lorenzo Livi, Daniele Giansanti

**Affiliations:** 1 Department of Allied Health Professions, Azienda Ospedaliero-Universitaria Careggi, 50134 Florence, Italy; andrea.lastrucci@unifi.it (A.L.); ioscan@aou-careggi.toscana.it (N.I.); wandaely@aou-careggi.toscana.it (Y.W.); barraa@aou-careggi.toscana.it (A.B.); riccire@aou-careggi.toscana.it (R.R.); 2 Neuroradiology Unit, Department of Radiology, Careggi University Hospital, 50134 Florence, Italy; giorgiobusto87@gmail.com; 3 Centro TISP, Istituto Superiore di Sanità, via Regina Elena 299, 00161 Rome, Italy; mirko.rossi@iss.it (M.R.); antonia.pirrera@iss.it (A.P.); 4 Medical Oncology, Santa Maria delle Croci Hospital, Ravenna AUSL, 48121 Rome, Italy; ilaria.morelli2@auslromagna.it; 5 Department of Experimental and Clinical Biomedical Sciences “M. Serio”, University of Florence, 50134 Florence, Italy; isacco.desideri@unifi.it (I.D.); lorenzo.livi@unifi.it (L.L.)

**Keywords:** radiology, radiotherapy, pain, anxiety, distress, virtual reality, augmented reality, mixed reality, extended reality

## Abstract

**Background/Objectives**: Pain, anxiety, and distress are common yet frequently insufficiently managed issues for patients undergoing radiology and radiotherapy procedures. Immersive technologies, including virtual reality (VR), augmented reality (AR), and mixed reality (MR), are emerging as innovative non-pharmacological approaches to alleviate such burdens through engaging interventions. This review, combining scoping and narrative methodologies, seeks to examine the current application, efficacy, and integration of these technologies to enhance patient care and wellbeing within diagnostic and oncological environments. **Methods**: Employing a mixed scoping and narrative review approach, this study conducted a systematic search of PubMed, EMBASE, Scopus, and Web of Science databases (no date restrictions—search included studies up to May 2025) to identify relevant studies utilizing VR, AR, MR, or XR for mitigating pain, anxiety, or distress in patients undergoing radiology or radiotherapy. Two independent reviewers selected eligible papers, with data extracted systematically. The narrative analysis supplemented the scoping review by providing contextual insights into clinical relevance and technological challenges. **Results**: The screening process identified 76 articles, of which 27 were assessed for eligibility and 14 met the inclusion criteria. Most studies focused on oncology and primarily employed VR as the immersive technology. VR has shown promising effects in reducing anxiety and pain—particularly during radiotherapy sessions and invasive procedures—and in supporting patient education through engaging, immersive experiences, making it a valuable approach meriting further investigation. Patient acceptance was notably high, especially among those with elevated distress levels. However, findings in radiology were less consistent, likely due to shorter procedure durations limiting the effectiveness of VR. The variability in outcomes highlights the importance of tailoring immersive interventions to specific procedures and patient needs. The narrative component identified key barriers, such as regulatory hurdles, standardization issues, and implementation challenges, that need addressing for broader clinical adoption. **Conclusions**: Immersive digital therapeutics are evolving from preliminary research tools toward more structured incorporation into clinical practice. Their future success relies on harmonizing technological advancements with patient-focused design and robust clinical evidence. Achieving this will require collaborative efforts among researchers, industry stakeholders, and healthcare providers. The integration of scoping and narrative review methods in this study offers a comprehensive perspective on the current landscape and informs strategic directions for advancing immersive technologies in radiology and radiotherapy.

## 1. Introduction

### 1.1. Background

Pain [1], anxiety [2], and distress are pervasive and complex challenges for patients throughout diagnostic and therapeutic care [1,2,3]. Often seen as secondary, these interrelated experiences deeply affect multiple dimensions of wellbeing. Pain, acute or procedural, is not only sensory but also emotional and cognitive, worsened by anticipation and uncertainty. Anxiety, especially pre-procedural, increases pain perception and reduces patients’ calm engagement with care [4,5]. This creates a reinforcing cycle where fear amplifies discomfort and vice versa, undermining resilience and tolerance. Distress overlaps but is broader, including situational unease to severe affective reactions like despair or intrusive thoughts, which can evolve into long-term psychological sequelae resembling PTSD, depression, or chronic anxiety, impacting quality of life and social function [6]. Clinically, unmanaged pain and distress worsen adherence, delay recovery, reduce success rates, and increase sedative use, risking vulnerable groups and deterring follow-ups, thereby harming outcomes. Despite awareness, care often focuses on pharmacological symptom control, neglecting the emotional and cognitive burdens accompanying medical care [7]. This gap calls for integrated, multidisciplinary approaches addressing both physical pain and psychological suffering. Specifically, in medical imaging, interventional cardiology, and radiotherapy, patients face unique challenges, heightening pain, anxiety, and distress. MRI requires prolonged immobilization in confined spaces, triggering claustrophobia and vulnerability, especially in anxious or traumatized patients [8]. CT and PET scans carry the emotional burden of “scanxiety”—fear of bad results—peaking during waiting times and disrupting sleep and concentration [9]. Interventional cardiology procedures, done under conscious sedation, expose patients to invasive sensations and unfamiliar equipment, provoking procedural pain, fear of complications, and existential anxiety. The clinical setting may cause disempowerment, leading to avoidance or emotional withdrawal, especially in those with low health literacy or social support [10]. Radiotherapy involves repeated, prolonged sessions with immobilization devices that can trigger panic and physical side effects that erode resilience over time. The solitary nature of treatments reinforces isolation and helplessness [11,12]. Across these contexts, unmanaged pain and distress contribute to incomplete procedures, increased sedation, and treatment avoidance, while triggering stress responses (cortisol elevation, tachycardia, and immune changes) that may affect healing and treatment efficacy. Psychological suffering may persist, reducing willingness for future care. Thus, technical skill alone is insufficient, and care models must integrate emotional, cognitive, and existential patient needs. Innovative complementary approaches gaining attention include mindfulness, which reduces anticipatory anxiety and decouples pain from catastrophic thoughts by fostering present-moment, non-judgmental awareness [13,14]. Neuroimaging confirms mindfulness modulates brain areas tied to threat and emotion [14]. Cognitive-behavioral therapy (CBT) helps patients reframe maladaptive thoughts and teaches coping strategies to regain control, proven effective in oncology and cardiology to reduce anxiety and improve adherence [15]. Music therapy reduces sympathetic arousal and activates brain reward circuits, enhancing safety feelings, especially when personalized [16]. Progressive muscle relaxation and guided imagery modulate autonomic nervous system activity, reduce muscle tension, and promote parasympathetic responses, effective in diverse populations, including children and adults undergoing invasive procedures. Biofeedback offers real-time monitoring of physiological signals, empowering patients to self-regulate anxiety and pain responses, useful in panic or pain flare episodes. Together, these approaches form a holistic, patient-centered care architecture fostering resilience and therapeutic engagement, especially important for vulnerable groups where sedation risks are higher. Digital therapeutics—app-based CBT, virtual coaching, and wearable stress monitors—expand psychosocial support accessibility before, during, and after procedures, enhancing behavioral interventions in real life [17]. Pre-procedure mobile CBT reduces anxiety and improves tolerance, while post-treatment digital coaching supports recovery and emotional coping. Immersive technologies—virtual reality (VR), augmented reality (AR), and mixed reality (MR)—are increasingly integrated into care workflows [18,19]. These use advanced graphics, interactivity, and multisensory feedback to alter pain, time, and threat perception [20]:

- VR fully immerses patients in calming or distracting environments, reducing procedural pain by over 30% in trials, notably in burns, chemotherapy, and MRI [21].
- AR overlays digital content on the real world, educating patients and reducing anticipatory fear, useful in pediatric and cognitively impaired populations.
- MR blends virtual and real elements, enabling interaction responsive to movement or biometrics, enhancing presence and agency in rehabilitation and pain management.

These technologies rewire attention, modulate pain via gate control, and shift emotions through embodied simulation, empowering patients beyond passive roles. Clinically, immersive therapies reduce sedative and opioid needs, shorten procedure times via improved cooperation, and stabilize vital signs during interventions [22,23]. Their adoption requires attention to infrastructure, usability, and equity but promises to transform medical sensory and emotional experiences.

### 1.2. Context, Rationale, and Purpose of the Mixed Scoping and Narrative Review

#### 1.2.1. Context and Rationale

The integration of immersive technologies, such as augmented reality (AR), mixed reality (MR), and virtual reality (VR), into radiology and radiotherapy represents a classic and multifaceted theranostic challenge—that is, the simultaneous management of diagnostic and therapeutic processes to optimize patient outcomes. Theranostics fundamentally involves the combination of diagnostic imaging and targeted therapeutic interventions, aiming to tailor treatments based on precise diagnostic information while addressing patient-centered needs throughout the care pathway.

In radiology and radiotherapy, this dual role is particularly pronounced. On one hand, these disciplines rely on highly accurate diagnostic and therapeutic procedures that are often invasive or anxiety-provoking for patients. On the other hand, they require effective management of associated symptoms, such as pain, anxiety, and distress, which can significantly impact patient adherence, satisfaction, and ultimately, treatment efficacy.

This intersection between diagnosis and therapy creates a complex clinical landscape where patient experience and procedural effectiveness are tightly interwoven. Immersive technologies, such as AR, MR, and VR, offer innovative solutions capable of bridging this gap by providing engaging, non-pharmacological interventions that can reduce pain and anxiety while supporting procedural success. However, their dual diagnostic–therapeutic potential also introduces challenges in terms of clinical validation, regulatory approval, and integration into existing care pathways.

Given these complexities, it is crucial to systematically evaluate the current evidence surrounding the use of immersive technologies for managing procedural symptoms in radiology and radiotherapy.

#### 1.2.2. Purpose of the Mixed Scoping and Narrative Review

Given the rapidly evolving and heterogeneous nature of the literature surrounding these technologies, a review approach that combines both scoping and narrative methodologies is particularly suited to the task. The scoping component enables a comprehensive mapping of existing studies, encompassing diverse immersive tools, clinical applications, and measured outcomes. This broad overview is essential to capture the multidisciplinary and innovative landscape of immersive digital therapeutics, where study designs and intervention protocols vary widely.

Simultaneously, the narrative component provides a critical synthesis and contextual analysis, enabling deeper exploration of complex issues, such as the theranostic implications, regulatory challenges, clinical integration pathways, and patient-centered design considerations. This interpretive layer helps bridge the gap between evidence mapping and actionable insights, fostering a nuanced understanding of how immersive technologies can be effectively implemented to optimize care in radiology and radiotherapy.

Therefore, the purpose of this mixed scoping and narrative review is to systematically explore and critically evaluate the current state of AR, MR, and VR applications for managing pain, anxiety, and distress in radiology and radiotherapy. By identifying key developments, gaps, and challenges, this review aims to inform future research, guide clinical implementation strategies, and support the broader integration of immersive digital therapeutics as effective adjuncts in theranostic care pathways—ultimately advancing holistic patient-centered oncology and diagnostic imaging.

## 2. Materials and Methods

This study adopted a hybrid methodological approach that combined the breadth and mapping power of a scoping review with a narrative synthesis of emerging patterns, themes, and interpretive insights. The scoping component followed the PRISMA-ScR guidelines and the Arksey and O’Malley framework [24,25], ensuring methodological transparency and reproducibility in study identification, selection, and data extraction. Meanwhile, the narrative layer was used to integrate and contextualize findings, offering a coherent storyline that could support critical reflection on the role of immersive technologies in pain and anxiety management during radiological and radiotherapy procedures.

### 2.1. Design and Search Strategy

In accordance with the Preferred Reporting Items for Systematic Reviews and Meta-Analyses extension for Scoping Reviews (PRISMA-ScR) [24] and the Arksey and O’Malley framework [25], a comprehensive electronic literature search was meticulously planned and conducted to identify relevant full-text original studies. These studies focused specifically on the application of immersive technologies—namely, virtual reality (VR), augmented reality (AR), mixed reality (MR), and extended reality (XR)—aimed at managing pain and anxiety in patients undergoing radiological examinations or radiotherapy treatments.

The search spanned multiple major biomedical and scientific databases, including PubMed, EMBASE, Scopus, and Web of Science, covering all available literature from the inception of each database up to May 2025. This broad temporal scope was chosen to ensure the capture of both early foundational studies and the most recent advances in immersive technologies applied to oncology and radiology contexts.

To optimize retrieval, detailed and tailored search strategies were developed and adapted for each database, balancing sensitivity and specificity. These strategies, including keywords, Boolean operators, and filters, are fully documented and can be found in Table S1 in the Supplementary Material for transparency and reproducibility.

Studies were selected based on explicit inclusion criteria to maintain focus and relevance:

(a) Publications had to be written in English.
(b) Investigations needed to assess the use of VR, AR, MR, or XR explicitly for reducing pain, anxiety, or psychological distress.
(c) The patient population had to involve individuals undergoing diagnostic or therapeutic radiological procedures, including imaging or radiotherapy.
  This targeted scope was designed to address a clinically meaningful intersection between immersive technologies and patient-centered outcomes in oncology and diagnostic settings.

Conversely, studies were excluded if they did not meet one or more of these criteria. Additional exclusions applied to non-peer-reviewed sources, such as conference abstracts, proceedings, and non-full-text papers, given their limited methodological detail and preliminary nature, which could compromise the quality of synthesis.

Regarding study types, both primary research (original clinical or experimental studies) and secondary research (systematic reviews, meta-analyses, and other evidence syntheses) indexed in the selected databases were deemed eligible, consistent with the broad mapping aims of scoping reviews and in line with the classification of medical research types, as outlined in [26].

Importantly, as this investigation constitutes a scoping review rather than a systematic review, no formal assessment of risk of bias or study quality was undertaken. This methodological choice is consistent with the guidance provided by Arksey and O’Malley [25], which highlights that scoping reviews aim primarily to map the breadth and nature of available evidence rather than to critically appraise the validity or robustness of individual studies. The checklist used to ensure adherence to PRISMA-ScR standards and review rigor is presented in Table S2 in the Supplementary Material.

A mixed (hybrid) approach was adopted, combining the broad exploratory mapping typical of a scoping review with a narrative synthesis component. Such integration enabled not only the identification and classification of studies, but also an interpretive discussion of emerging themes, trends, and gaps in the literature. The narrative element adds depth by contextualizing quantitative findings within patient experience, technological characteristics, and clinical applicability, offering a richer, multi-dimensional understanding of immersive technologies for pain and anxiety management in radiology and radiotherapy.

This structured and transparent approach facilitated a comprehensive overview of the literature, identifying key trends, gaps, and characteristics of research on immersive technologies in pain and anxiety management within radiology and radiotherapy, thus providing a solid foundation for subsequent qualitative (narrative) and quantitative synthesis.

### 2.2. Study Selection and Data Extraction

After completing the literature search, all retrieved references were systematically imported into Mendeley Reference Manager (version 2.120.1) to ensure organized and efficient management of the bibliographic data. The first step involved the identification and removal of duplicate records, which was initially conducted using Mendeley’s automatic duplicate detection function. This automated process allowed for rapid preliminary cleansing of the dataset; however, recognizing the limitations of software-based duplicate detection, a subsequent manual review was meticulously performed to confirm that all duplicates had been accurately identified and completely removed. This two-tiered approach minimized the risk of inadvertent inclusion of redundant records, which could bias the review findings.

Following the de-duplication process, the remaining studies underwent a rigorous screening phase conducted independently by two reviewers (A.L. and D.G.). This phase involved a preliminary screening of titles and abstracts with the primary aim of excluding articles that were clearly irrelevant or off-topic based on predefined eligibility criteria. By employing a dual independent review, the process ensured objectivity and minimized individual bias in study selection.

For those studies deemed potentially relevant at the title and abstract stage, the full-text articles were subsequently obtained and thoroughly assessed for eligibility by the same pair of reviewers. During this stage, detailed evaluation against the inclusion and exclusion criteria was performed to determine the suitability of each study for final inclusion. In cases where disagreements arose between reviewers regarding eligibility or interpretation of inclusion criteria, a consensus-based resolution process was employed. This involved open discussion and deliberation until full agreement was reached, thereby enhancing the reliability and transparency of the study selection.

Once eligible studies were confirmed, data extraction was independently carried out by both reviewers using a purpose-built electronic database specifically designed for this scoping review. This electronic system streamlined the collection, tracking, and updating of key study variables and outcomes, facilitating accurate and efficient data management. Extracted data included comprehensive details, such as study characteristics, intervention types, patient populations, outcomes measured, and main findings.

To ensure the highest level of data integrity and validity, the final extracted dataset underwent a thorough cross-check and review by the entire research team. This collective review process aimed to identify any inconsistencies, resolve discrepancies, and validate the completeness and accuracy of the dataset before proceeding to synthesis and analysis. The collaborative effort strengthened the overall methodological rigor of the review and provided confidence in the reliability of the results presented.

### 2.3. Analytic Approach

Following a comprehensive data extraction process, all relevant information from the selected studies was systematically summarized and collectively reviewed by the research team. To ensure consistency and facilitate detailed comparison across studies, a standardized data extraction form was developed and implemented using Microsoft Excel (Redmond, Washington, DC, USA). This tool enabled the organized consolidation of a wide range of study details in a clear, accessible format, facilitating both data management and collaborative analysis.

The extraction process focused on capturing essential characteristics of each study, such as the author(s) and year of publication, which helped situate the research within a chronological and scientific context. Careful attention was paid to study design and methodology to understand the rigor and nature of the evidence provided, alongside sample size to assess the scale and statistical robustness of the findings. Furthermore, the clinical setting or treatment location was recorded to evaluate the applicability and potential generalizability of the results across different healthcare environments.

A central aspect of the extraction was the precise identification and classification of the immersive technology employed in each study. This included differentiating among virtual reality (VR), augmented reality (AR), mixed reality (MR), and extended reality (XR), which allowed the mapping of the evolving technological landscape and facilitated identification of specific trends, gaps, or underexplored areas within the field. Additionally, study objectives were documented in detail to clarify research questions and intended outcomes, complemented by the main results and conclusions to capture core findings and their broader clinical or technological implications.

To effectively integrate the dual goals of breadth and depth characteristic of this hybrid review, two complementary analytic streams were undertaken:

- **Descriptive mapping (scoping component):**
  This involved systematic tabulation and summarization of key study attributes, including study designs, participant demographics, clinical contexts, types of immersive technologies used, and the targeted symptoms or outcomes, such as pain, anxiety, and psychological distress. This mapping functioned as a comprehensive inventory of available evidence, facilitating an understanding of the overall distribution, scope, and characteristics of the research landscape.
- **Narrative synthesis (interpretive component):**
  Beyond descriptive aggregation, the research team engaged in iterative, collaborative discussions to identify and explore emerging themes, patterns, and contextual nuances that emerged from the data. This qualitative synthesis examined factors influencing implementation and effectiveness, such as technology usability, patient acceptance, integration challenges within clinical workflows (e.g., compatibility of headsets with radiotherapy equipment), and population-specific preferences (e.g., pediatric versus adult patients). This interpretive process provided richer insights into the how and why certain immersive interventions succeeded or faced limitations and offered critical reflections to inform future research priorities and practical applications.

This dual analytic framework thus enabled the review to maintain methodological rigor and comprehensive coverage typical of scoping studies, while simultaneously providing interpretive depth and critical insight characteristic of narrative reviews. Such an approach enhanced the overall value of the synthesis, fostering a nuanced understanding of the current evidence and highlighting areas ripe for further investigation.

Ethical approval was not required for this study.

### 2.4. Assessment of Potential Bias

Because this work was designed as a mixed scoping and narrative review, rather than a systematic review with a quantitative meta-analysis, formal statistical tools typically used to assess publication bias—such as funnel plots, Egger’s tests, or quantitative sensitivity analyses—were not applicable. The primary objective of this review was to map and synthesize the breadth of available evidence and to identify emerging themes, gaps, and contextual factors, rather than to calculate pooled effect sizes.

Nonetheless, several methodological safeguards were implemented to reduce the potential for bias and to enhance the transparency and robustness of the findings. A comprehensive search strategy was developed and applied across multiple major biomedical and scientific databases (PubMed, EMBASE, Scopus, and Web of Science), with no restrictions on the start date and inclusion of all available literature up to May 2025. Search strategies were tailored for each database, balancing sensitivity and specificity, and are fully documented in the Supplementary Materials to ensure reproducibility.

To further limit bias, inclusion and exclusion criteria were defined a priori and applied consistently. All records underwent dual independent screening at both the title/abstract and full-text stages, with discrepancies resolved through consensus. This process minimized the influence of individual reviewer bias on study selection. Similarly, data extraction was carried out independently by two reviewers using a standardized template, followed by cross-verification to ensure accuracy and completeness.

Although no formal statistical assessment of publication bias was conducted, the narrative synthesis component included a qualitative appraisal of the heterogeneity in study designs, patient populations, intervention modalities, and outcome measures. This allowed the review to highlight patterns that may indirectly indicate areas of overrepresentation or underreporting, thereby offering insight into potential biases within the evidence base.

Future systematic reviews or meta-analyses focused on specific immersive technologies, patient groups, or clinical outcomes could complement this work by applying formal quantitative techniques, such as funnel plots, trim-and-fill methods, or sensitivity analyses. Combining such approaches with the broader mapping and contextual interpretation offered by the present review would yield a more comprehensive and multi-layered understanding of the role of immersive technologies in managing pain, anxiety, and distress during radiology and radiotherapy procedures.

## 3. Results

*Rationale for the Evidence Synthesis Structure*

This section is carefully organized to provide a rigorous and comprehensive synthesis of the evidence, enabling a clear, systematic understanding of current research trends, methodological rigor, and clinical applications of immersive technologies in oncology and radiology settings. The structure reflects a dual purpose: first, to map the scope and heterogeneity of the existing literature, and second, to deliver an insightful thematic analysis that elucidates critical dimensions shaping the field. This design ensures that readers gain both a broad overview and a nuanced interpretation of the evidence, supporting informed conclusions and identifying pathways for future inquiry.

- Section 3.1—Inclusion Criteria and Study Characteristics
  This foundational subsection establishes the context by detailing the selection criteria and summarizing the key attributes of the included studies. By describing the immersive technologies utilized—primarily virtual reality—and their clinical contexts, such as radiotherapy, diagnostic/interventional radiology, and pediatric oncology, alongside methodological elements like patient populations, sample sizes, and outcome metrics, this section lays crucial groundwork. It captures the diversity and complexity of the literature, framing the heterogeneity in interventions, study designs, and outcomes, which is essential for appreciating the scope and limitations of the current evidence base. This broad mapping corresponds to the scoping objective of the review, offering transparency and orientation.
- Section 3.2—Thematic Analysis of Key Dimensions
  Following this overview, the evidence is synthesized through five carefully delineated thematic areas derived from a detailed qualitative and quantitative analysis. It moves from descriptive mapping to thematic analysis, distilling the evidence into five high-impact domains—patient engagement, procedural efficiency, equity and access, methodological robustness, and personalization—each of which represents a pivotal determinant for successful implementation. This thematic architecture is designed to be both clinically intuitive and analytically rigorous, enabling the reader to identify not only “what works” but also “under what conditions” and “for whom.” These themes address critical facets of immersive technology applications in clinical oncology and radiology:
  ○ Section 3.2.1—Patient Engagement and Psychological Readiness
    Investigates how individual patient factors, such as motivation, baseline anxiety, and technological receptiveness, influence intervention effectiveness. This theme highlights the importance of tailoring immersive approaches to patient-specific psychological states to optimize therapeutic outcomes.
  ○ Section 3.2.2—VR and Procedural Efficiency
    Explores the impact of immersive technologies on clinical workflow and procedural dynamics, including duration and sedation needs, assessing their feasibility as adjuncts or alternatives to pharmacological interventions. This analysis addresses concerns about operational integration and patient comfort.
  ○ Section 3.2.3—Equity and Access
    Examines barriers related to digital literacy, accessibility, and usability, underscoring the risk of unequal benefits across diverse patient populations. It emphasizes strategies for inclusive implementation to ensure equitable access to immersive healthcare innovations.
  ○ Section 3.2.4—Methodological Limitations
    Highlights the considerable variability in study designs, sample sizes, and outcome measures, advocating for the development and adoption of standardized protocols. Addressing these methodological challenges is crucial for strengthening the evidence quality and reproducibility.
  ○ Section 3.2.5—Personalization and Precision
    Focuses on emerging trends toward tailoring immersive content to individual clinical and psychological profiles, aligning with precision medicine paradigms. This theme underscores the potential for customized digital interventions to enhance anxiety and pain management efficacy.

This thematic and interpretative synthesis not only organizes the diverse body of evidence into coherent, clinically relevant domains but also illuminates emerging research trends, practical implications, and critical knowledge gaps. By doing so, it bridges descriptive mapping with analytical insight, offering a narrative that informs both clinical practice and future research directions.

To support transparency and enable deeper inquiry, detailed analytical summary sheets for each included study are provided in the Supplementary Material, serving as an in-depth resource for interested readers.

Collectively, this structured approach captures the complex and promising role of immersive technologies in transforming oncologic and radiologic care, providing a robust foundation for advancing patient-centered applications and guiding systematic integration into clinical workflows.

### 3.1. Inclusion and Characteristics of the Studies

**Figure 1:**
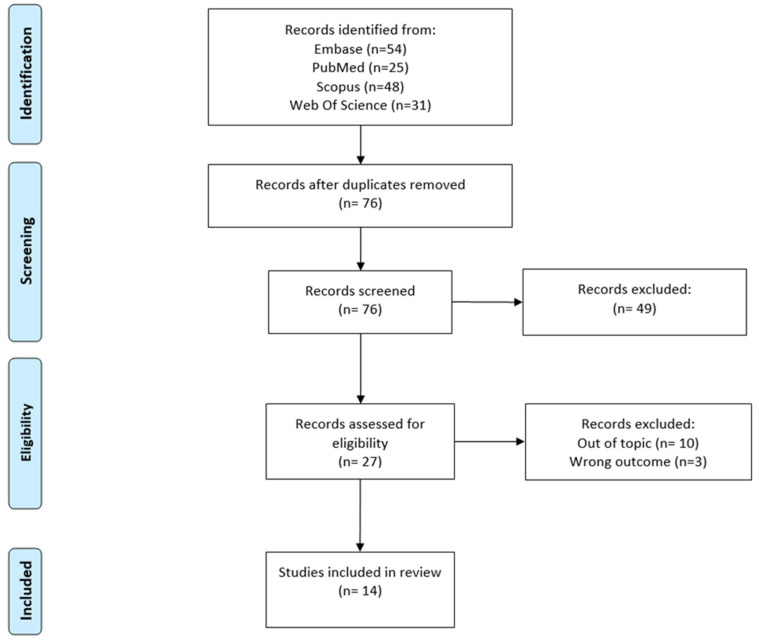
Flowchart of study selection.

A total of 14 articles met the inclusion criteria and were included in this scoping review [27,28,29,30,31,32,33,34,35,36,37,38,39,40]. Figure 1 shows the study selection process in the format of a PRISMA diagram.

All included articles in this review were published between 2018 and 2025. A summary of the evidence from these studies is presented in Table 1.

From the comprehensive analysis of the reviewed literature, it clearly emerges that virtual reality (VR) stands out as the predominant and most extensively studied immersive technology employed for the management of anxiety and pain in patients undergoing various diagnostic and therapeutic medical procedures. Among the different immersive modalities explored, VR has consistently attracted the greatest clinical and scientific attention due to its technological maturity, ease of integration into existing care pathways, and its capacity to provide a controlled, engaging, and personalized multisensory experience.

The widespread clinical interest in VR stems from its ability to act as a non-invasive, drug-free adjunct to conventional treatments, targeting the emotional and perceptual aspects of patient experience. Specifically, VR is increasingly being recognized for its potential to alleviate procedure-related distress, mitigate anticipatory anxiety, and reduce perceived pain intensity during medical interventions that are often experienced as uncomfortable or frightening.

Across the 14 studies examined, VR technology has been applied in a diverse array of clinical environments. Notably, it features prominently in radiotherapy settings (reported in 7 studies), in diagnostic and interventional radiology contexts (6 studies), and in pediatric oncology care (1 study). This broad applicability reflects VR’s flexibility and adaptability to different patient populations, procedural types, and phases of the care pathway—whether as a pre-procedural preparatory tool, a distraction technique during invasive procedures, or as a supportive element in chronic treatment regimens.

In these various applications, VR is most frequently deployed as a cognitive-behavioral tool designed to immerse patients in interactive, calming virtual environments that reduce focus on the external clinical stimuli. Such immersive content may include naturalistic landscapes, animated games, guided meditation modules, or simulation-based educational walkthroughs. These experiences serve to divert attention away from the medical procedure itself, thereby lowering the activation of stress-related physiological pathways and modulating pain perception through top-down neurological mechanisms.

Evidence from randomized controlled trials (RCTs)—the gold standard in clinical research—consistently supports the efficacy of VR in reducing both self-reported pain and anxiety scores, often without prolonging the overall duration of the procedure. For instance, Schaake et al. (2024) demonstrated that the application of VR during interventional radiology procedures—specifically during peripherally inserted central catheter (PICC) placements and thyroid fine-needle aspirations—resulted in statistically significant reductions of 1.74 points in pain and 1.60 points in anxiety on validated visual analog scales (VASs) [27]. These results are particularly relevant, as they demonstrate not only the effectiveness of VR but also its feasibility and efficiency within time-sensitive procedural workflows.

Similarly, Gullo et al. (2023) explored the use of a VR-based self-hypnosis application in patients undergoing endovascular interventions [28]. Their findings showed a substantial increase in the proportion of patients who responded to anxiety reduction: 76% of participants in the VR group were classified as responders, compared to 46% in the standard care group [28]. This underscores VR’s potential to empower patients by giving them active tools for managing their emotional state, thus promoting a greater sense of control and engagement during procedures.

In the context of radiotherapy, VR has shown to be particularly effective not only in reducing psychological burden but also in enhancing patient understanding of complex treatment regimens. For example, Gao et al. (2022) found that patients receiving chest radiotherapy who used VR exhibited significant improvements in both psychological (reduced anxiety and systolic blood pressure) and educational outcomes, with better comprehension of the therapeutic pathway [29]. Such findings suggest that VR may serve a dual role as both a therapeutic and educational interface, enhancing patient satisfaction, compliance, and trust in the treatment team.

The potential of VR in radiotherapy was further reinforced by the systematic review conducted by Grilo et al. (2023), which synthesized results from multiple studies and concluded that VR-based educational sessions were consistently associated with reductions in patient anxiety across different anatomical sites and phases of treatment [30]. These results support the broader integration of VR into patient onboarding and treatment preparation, particularly in contexts where complex technology and unfamiliar environments might provoke distress.

Beyond the immediate goals of reducing pain and anxiety, some studies reported additional benefits in patient-reported outcomes, including improvements in fatigue, engagement, and emotional wellbeing. Notably, Hernandez et al. (2025) investigated a cohort of lung cancer patients undergoing chemoradiotherapy and observed significant improvements in a triad of symptoms: anxiety, pain, and fatigue [31]. These findings open new possibilities for the use of VR not just as a periprocedural tool, but as a component of supportive oncology care aimed at improving quality of life during multimodal treatments.

Nevertheless, the evidence is not uniformly positive, and some studies reported neutral findings, suggesting that VR’s effectiveness is context dependent. For example, Jimenez et al. (2018) found no significant differences in anxiety reduction during breast radiotherapy when VR was used, highlighting that variables such as type of cancer, procedural duration, patient expectations, and content personalization may influence outcomes [32]. These inconsistencies underline the importance of refining intervention protocols and tailoring VR experiences to the specific needs and characteristics of each patient population.

In the field of diagnostic radiology, results remain heterogeneous. Bay et al. (2024) reported no meaningful impact of VR on pain levels during mammography, which may be due to the short duration and relatively low invasiveness of the procedure [33]. In contrast, Grange et al. (2024) found that VR contributed to significant reductions in both pain and anxiety during more complex interventional radiology procedures, supporting the idea that VR’s benefits are more pronounced during longer or more invasive treatments [34]. Similarly, Gonzalez et al. (2024) developed and tested a tailored VR application for MRI-guided radiotherapy in abdominal cancer patients and demonstrated meaningful anxiety reduction and improved patient readiness, particularly among those with high baseline distress [35].

Patient acceptability of VR interventions is consistently high across studies, with most participants reporting a positive experience and expressing interest in future use. For example, Wong et al. (2023) found that patients undergoing prostate cancer procedures, such as transperineal biopsies and fiducial marker implantations, showed high interest in using VR-based hypnosis to manage procedural anxiety and discomfort [36]. In the pediatric oncology setting, immersive VR applications were found to facilitate environmental adaptation, helping children become familiar with the radiotherapy room and easing the transition into treatment, as illustrated by Alanazi et al. (2022) [39].

Importantly, VR also holds potential as an alternative or complement to pharmacologic sedation, especially in procedures where sedation poses risks or is not readily available. Varnier et al. (2021) demonstrated that the use of VR during removal of uterovaginal brachytherapy applicators was as effective as standard sedation methods in controlling pain and anxiety [38]. In some cases, it even outperformed sedation in terms of patient satisfaction and cognitive clarity, suggesting that VR could play a role in promoting sedation-sparing protocols, improving recovery times, and minimizing side effects. Additional insights emerge when considering broader evidence on procedural support and analgesia. Cornelis et al. (2019) reviewed sedation and analgesia strategies in interventional radiology, emphasizing the growing interest in non-pharmacological adjuncts like VR to minimize drug-related risks and enhance patient cooperation [37]. Building on this, Perenic et al. (2023) explored the application of immersive VR during MRI-guided prostate biopsies and reported a significant reduction in pain scores and improved patient comfort [40]. These findings underscore the versatility of VR not only in radiotherapy and pediatric oncology but also in complex diagnostic contexts involving real-time imaging. Collectively, the studies suggest that immersive technologies may serve as viable complements—or even alternatives—to traditional sedation practices, supporting a shift toward patient-centered, minimally invasive care pathways.

Overall, the growing body of evidence supports the integration of virtual reality (VR) as a promising, safe, and effective adjunct in both radiology and radiotherapy clinical settings. VR interventions appear particularly effective in pre-treatment education and procedures associated with acute discomfort. For example, oncology patients scheduled for MRI-guided radiotherapy who used an interactive VR application demonstrated significant reductions in pre-treatment anxiety and improved procedural understanding (Ref. [35]). Similarly, women undergoing brachytherapy applicator removal reported marked decreases in pain and anxiety when immersed in calming VR environments during the procedure (Ref. [38]). In other contexts, such as interventional radiology and pediatric inpatient care, VR shows potential benefits—enhancing patient comfort, mood, and distraction from pain—but the evidence remains largely preliminary. Narrative reviews highlight VR as a possible adjunct to sedation and analgesia in interventional radiology, yet robust comparative data are limited (Ref. [37]), while pilot studies in pediatric oncology inpatients indicate improved quality of life and emotional wellbeing, though generalizability is constrained by small sample sizes and exploratory designs (Ref. [39]).

Despite these promising results, variability in study findings, methodological heterogeneity, and limited sample sizes underscore the need for further large-scale, well-designed, and standardized investigations. Future research should aim to optimize VR protocols, evaluate long-term outcomes, and delineate the clinical scenarios where VR can most effectively enhance patient-centered care.

### 3.2. Immersive Technologies in Clinical Settings: Key Themes and Insights

Immersive technologies, especially virtual reality (VR), are increasingly used to reduce patient anxiety, pain, and distress during diagnostic and therapeutic procedures. This section highlights key themes affecting their clinical success: patient engagement and psychological readiness, procedural efficiency and workflow, equity and access, methodological research challenges, and personalized adaptive interventions. Together, these themes reveal the complex but promising role of immersive tech in healthcare.

#### 3.2.1. Patient Engagement and Psychological Readiness

Patient engagement and readiness are crucial for VR’s success in oncology. Effectiveness depends on cognitive openness, emotional willingness, and mental focus. VR is an active interaction, not a mere distraction. For example, Gullo et al. [28] found that 76% of patients using VR self-hypnosis had reduced anxiety, versus 46% in standard care. Wong et al. [36] showed patients with higher baseline anxiety and pain had greater interest and acceptance of VR hypnosis, underlining the role of psychological state. Preparatory methods like motivational interviewing and education improve readiness. Feeling in control during VR interventions further reduces distress. Thus, patient engagement should be a core design element, with tailored approaches to optimize outcomes.

#### 3.2.2. VR and Procedural Efficiency: Time, Workflow, and Sedation

Concerns exist about VR increasing procedure time, but evidence shows it does not prolong interventions and may improve efficiency. Schaake et al. [27] reported no change in duration during VR use in interventional radiology, despite anxiety and pain reductions. Varnier et al. [38] observed VR provided analgesic and anxiolytic effects comparable or superior to sedation during brachytherapy applicator removal, enabling sedation reduction or avoidance. Less sedation reduces risks and workload on anesthesia and monitoring. Calm patients cause fewer interruptions, enhancing workflow. VR’s role in sedation-free protocols may optimize resources, lower costs, and improve safety, supporting more patient-centered oncology care.

#### 3.2.3. Equity and Access in Immersive Care Models

VR’s promise is limited by disparities in digital literacy, cultural acceptance, language, cognition, age, and socioeconomic factors. Pediatric patients respond well with coaching [39], while elderly or cognitively impaired patients face barriers like navigation difficulties and discomfort. Resource-poor institutions may lack hardware and support, risking inequities that favor younger, wealthier, urban patients. To counter this, VR platforms should be accessible: simplified interfaces, multilingual and culturally sensitive content, low-cost devices, and training for staff and patients. Policy and funding must promote equitable distribution. Addressing these challenges ensures immersive oncology care benefits all patients rather than widening gaps.

#### 3.2.4. Limitations and Methodological Heterogeneity in VR Studies

While virtual reality (VR) research in oncology shows promising trends in anxiety and pain management, the overall evidence base is constrained by substantial methodological heterogeneity and limitations that hinder definitive conclusions and clinical translation. Studies vary widely in intervention design, ranging from passive VR distraction to interactive self-hypnosis or educational modules, creating challenges in comparing outcomes directly. Patient populations differ by cancer type, treatment phase, age, and baseline psychological profiles, while sample sizes are often small, reducing statistical power and generalizability. For example, Gonzalez et al. [35] and Gao et al. [29] reported significant anxiety and pain reductions with VR interventions, whereas Jimenez et al. [32] found no statistically meaningful effects, potentially due to differences in VR content, duration, or patient engagement strategies. The lack of standardized outcome measures further complicates meta-analyses—studies employ varied anxiety scales, pain assessment tools, and physiological markers without uniformity. Additionally, many trials suffer from inadequate blinding or absence of sham VR controls, raising the risk of placebo effects and observer bias. Reporting inconsistencies, such as insufficient detail on VR hardware specifications or intervention protocols, reduce reproducibility. To advance the field, future investigations must adopt rigorous, standardized research methodologies: large, multicenter randomized controlled trials with appropriate control groups, uniform, validated outcome measures, transparent reporting of VR content and delivery parameters, and exploration of dose–response effects and long-term clinical outcomes. Only through such systematic approaches can immersive VR’s true clinical efficacy, optimal application parameters, and best practices be established in oncology care.

#### 3.2.5. Toward Personalized Immersive Oncology: Matching VR Content to Patient Profiles

The advancement of virtual reality (VR) enables personalized, adaptive oncology interventions tailored to patient and clinical factors. VR effectiveness varies by cancer type, treatment phase, prior tech familiarity, psychological state, and symptoms. For example, educational VR before radiotherapy reduces anxiety and improves readiness [29], while VR during invasive procedures serves mainly as a distraction to ease pain and distress [27,34]. Future VR may use real-time physiological monitoring and AI to dynamically adjust content, shifting toward “immersive precision medicine.” This would tailor interventions to emotional, cognitive, and clinical needs, integrating multimodal sensory input and user interaction to maximize benefits and reduce discomfort. Such adaptive VR could evolve from a distraction tool to a core therapy, improving quality of life and treatment adherence.

## 4. Discussion

*Rationale for the Discussion Structure*

The Discussion Section is intentionally structured into four thematic sections, followed by a dedicated limitations section, to maximize clarity, maintain logical progression, and ensure that the review’s objectives are addressed with both depth and breadth. This approach enables readers to follow a clear analytical trajectory: from interpreting the findings in a focused clinical context, to situating them within the conceptual framework of digital therapeutics (DTx), exploring the market and translational potential, and finally considering integration into established therapeutic models. The limitations section provides transparency and frames priorities for future research.

- Section 4.1—Clinical Interpretation of Findings
  This section provides a critical synthesis of the evidence, emphasizing the clinical significance of immersive technologies in mitigating anxiety, pain, and distress, particularly in radiology and radiotherapy settings. By connecting study outcomes to patient-centered benefits, it contextualizes how immersive interventions operate across different stages of care and modalities (e.g., radiotherapy preparation vs. intra-procedural distraction). The discussion goes beyond descriptive reporting to explore mechanisms of action, moderating factors, and the potential for personalization—linking empirical evidence to real-world clinical decision-making.
- Section 4.2—Positioning Within Digital Therapeutics (DTx)
  Placing immersive technologies within the DTx framework provides conceptual and regulatory clarity. This section examines the transition from “distraction-based adjuncts” to “clinically validated digital interventions,” drawing on definitions and standards from regulatory authorities, such as the FDA and EMA. It outlines the requirements for DTx designation, including evidence from randomized controlled trials, standardized treatment protocols, and post-market surveillance. This positioning is essential to elevate immersive technologies from experimental or niche applications to scalable, evidence-based medical solutions with defined therapeutic claims.
- Section 4.3—Market Growth and Translational Potential
  Immersive digital therapeutics are experiencing rapid commercial expansion, driven by increasing demand for non-pharmacological treatments for pain and anxiety. This section situates the clinical promise within the broader market landscape, considering technology readiness, cost-effectiveness, industry investment trends, and integration into existing healthcare systems. By addressing both drivers (e.g., patient demand and technological maturity) and barriers (e.g., interoperability and data privacy), it bridges the gap between research findings and real-world adoption.
- Section 4.4—Integration with Established Psychological Therapies
  Immersive technologies hold the potential to enhance and extend the impact of established interventions, such as cognitive-behavioral therapy (CBT) and mindfulness. This section discusses the theoretical and practical basis for such integration, considering factors like engagement, adherence, and transfer of learning to daily life. It also identifies systemic challenges that must be addressed—such as the lack of standardized clinical guidelines, uncertainty in reimbursement models, and the need for multicenter validation—to enable widespread adoption within multidisciplinary care pathways.
- Section 4.5—Limitations
  This section highlights the strengths of our mixed scoping and narrative review approach, while acknowledging typical limitations, such as the absence of formal bias assessments and sensitivity analyses due to its non-systematic design, and the re-striction to English-language studies, which future research could address.

By adopting this structure, the review not only organizes the discussion in a coherent and accessible way but also ensures that each dimension—clinical impact, conceptual positioning, market relevance, and therapeutic integration—is addressed systematically. This framework mirrors the translational pathway from bench to bedside, reinforcing the review’s value for clinicians, researchers, policymakers, and industry stakeholders seeking to understand both the current state and future trajectory of immersive digital therapeutics in oncology-related imaging and treatment contexts.

### 4.1. Interpretation of Findings and Clinical Relevance

In this study, a mixed approach combining scoping and narrative synthesis was adopted to comprehensively map the existing literature while providing interpretative insights. This methodology enabled capturing both the broad landscape of immersive technology applications in pain and anxiety management and the nuanced understanding of their clinical relevance, advantages, and limitations.

Immersive technologies, particularly VR, are emerging as innovative tools to support patients undergoing radiology and radiotherapy procedures, which are often associated with significant anxiety, pain, and emotional distress. VR’s capacity to create engaging, multisensory environments offers a promising approach to alleviate these burdens by providing distraction, education, and emotional support. This part of the discussion synthesizes current evidence on VR’s effectiveness in reducing anxiety and pain, its role as an educational tool, patient acceptability, and the limitations that temper its clinical adoption.

#### Summary of Findings

The literature reviewed indicates that virtual reality (VR) interventions within radiotherapy settings are associated with reductions in patient anxiety and, in some cases, reduced pain perception. Multiple high-quality studies, including those by Gao et al. [29], Hernandez et al. [31], and Grilo et al. [30], provide evidence that immersive VR-based educational sessions reduce emotional distress and improve patients’ understanding of complex and prolonged radiotherapy treatment regimens. This dual functionality of VR—as both an emotional support tool and an interactive educational platform—is particularly valuable in radiotherapy, where patients face long treatment durations combined with psychological burdens, such as uncertainty, fear, and stress. Immersive VR sessions allow patients to become familiar with the treatment environment, procedures, and expected sensations in a controlled, structured manner, reducing anticipatory anxiety and fostering engagement and participation in their care.

Conversely, evidence regarding VR applications in radiological procedures is more heterogeneous and context dependent. For instance, studies by Schaake et al. [27] and Gullo et al. [28] demonstrate reductions in anxiety and pain during some interventional radiology procedures, such as vascular access or catheter placements, where procedural invasiveness and patient discomfort are higher. These findings suggest that VR’s distraction and relaxation effects can modulate pain perception and psychological distress in settings with heightened procedural stress. However, research by Bay et al. [33] indicates limited benefit in procedures like mammography, which are shorter and less invasive, producing less anxiety or pain initially. These contrasting outcomes highlight the importance of procedural context—duration, invasiveness, and patient experience—when evaluating VR’s potential impact.

*Strengths and Clinical Benefits*

VR shows strong potential in invasive, lengthy, or potentially painful procedures, such as biopsies, catheter insertions, or brachytherapy applicator removals, as shown by Schaake et al., Varnier et al., and Perenic et al. [40]. In these scenarios, VR can complement sedation or reduce the need for pharmacologic sedation by diverting attention from discomfort and activating pain modulation pathways. This non-pharmacologic approach helps minimize sedation-related risks and supports faster post-procedural recovery.

In pediatric oncology, VR benefits are particularly observable. Alanazi et al. [39] highlighted VR’s role in helping young patients become familiar with intimidating treatment environments and complex equipment, reducing anticipatory anxiety and supporting treatment compliance. Early positive experiences may contribute to better long-term coping strategies and overall adherence.

A key aspect of VR is its educational capacity. Immersive VR experiences facilitate “therapeutic literacy” by allowing patients to visualize and interact with 3D representations of radiotherapy procedures, making abstract or difficult-to-understand concepts more tangible. This improved comprehension reduces uncertainty, supports confidence, and empowers patients to take an active role in care management (Gao et al. [29], Grilo et al. [30], and Gonzalez et al. [35]). Compared to verbal or written instructions, VR’s multisensory engagement improves knowledge retention and emotional preparedness, which can translate into better clinical outcomes and patient satisfaction.

Patient acceptance is a key determinant of VR’s success. Wong et al. [36] observed that patients with higher baseline distress are more motivated to engage with VR interventions, highlighting the need for personalized, adaptive VR content and session durations tailored to individual needs. The novelty, interactivity, and immersive nature of VR support psychological resilience, helping patients cope with the emotional challenges of cancer treatment.

To maximize usability and therapeutic benefit, clinicians must consider patient-specific factors, such as age, cognitive ability, technological familiarity, and prior exposure to immersive tools. Tailoring VR interventions to these variables optimizes comfort, minimizes barriers, and ensures equitable access across diverse patient populations.

*Limitations and Gaps*

Despite these encouraging results, several important limitations must be acknowledged, which currently hinder the ability to draw definitive conclusions about VR’s effectiveness in oncology care. A prominent challenge is the methodological heterogeneity among studies, including variability in intervention protocols, types of VR content delivered, timing of the interventions (pre-, during, or post-procedure), outcome measures used, and the clinical populations studied. This heterogeneity complicates cross-study comparisons and meta-analytic synthesis, reducing the generalizability of findings.

Small sample sizes and underpowered trials are frequent, limiting statistical robustness and the detection of clinically meaningful effects. Moreover, some studies, such as that of Jimenez et al. [32], report no significant anxiety reduction in breast radiotherapy patients, and Bay et al. [33] found no pain relief during mammography, suggesting that VR’s impact may be highly context-specific and influenced by patient and procedural characteristics. These mixed outcomes highlight the need for standardized intervention protocols and well-designed randomized controlled trials with sufficient sample sizes to clarify VR’s mechanisms of action and identify which patient subgroups derive the most benefit.

Technological and practical barriers further limit the widespread implementation of VR in clinical settings. These include the costs and maintenance of VR hardware, clinician training requirements, potential workflow disruptions, and the need for user-friendly interfaces adaptable to various healthcare environments. Overcoming these challenges requires coordinated efforts involving technical developers, clinicians, and healthcare administrators to integrate VR solutions seamlessly into routine oncology care.

### 4.2. Immersive Technologies in Radiology and Radiotherapy: Defining Their Role Within Digital Therapeutics

While growing evidence supports the clinical potential of immersive technologies, such as virtual reality (VR), augmented reality (AR), and mixed reality (MR), in reducing anxiety, pain, and distress during radiological and radiotherapeutic procedures, their exact classification within the evolving framework of digital therapeutics (DTx) remains somewhat ambiguous. Unlike generic digital health apps focused on wellness or monitoring, DTx delivers clinically validated, evidence-based interventions, either as standalone treatments or adjuncts to conventional care, via software platforms targeting specific medical conditions with rigorous evaluation of efficacy, safety, and regulatory compliance [41].

When developed with clearly defined therapeutic protocols, supported by robust clinical trials and regulatory standards, immersive technologies go beyond distraction or relaxation tools. VR, AR, or MR platforms can function as true therapeutic agents, providing measurable clinical benefits beyond symptom alleviation. This transition—from entertainment or engagement tools to evidence-based treatments—aligns with evolving DTx definitions promoted by groups like the Digital Therapeutics Alliance (DTA). The growing acceptance is reflected in regulatory milestones, such as the FDA clearance of VR-based interventions for chronic pain and anxiety, exemplified by *RelieVRx* by AppliedVR, authorized in 2021 as a prescription digital therapeutic for chronic lower back pain, underscoring VR’s legitimacy as a therapeutic modality [42,43].

However, in radiology and radiotherapy, most immersive applications remain exploratory or small-scale, with limited integration into standard clinical pathways. This limited adoption arises from several factors: a lack of regulatory clarity about valid “therapeutic claims” complicates formal recognition and reimbursement, the challenge of conducting large multicenter randomized controlled trials in acute, variable clinical environments, and a gap in awareness among healthcare professionals, who may confuse DTx with wellness apps, causing hesitancy to adopt these technologies routinely [44,45].

Bridging this gap requires multifaceted change in both research and institutional perspectives. Research must evolve toward large-scale, rigorous studies applying standards used for pharmaceuticals and established DTx. Simultaneously, healthcare institutions and providers must acknowledge immersive technologies, when designed for therapeutic purposes, as legitimate medical interventions with measurable outcomes. This recognition would support their integration into existing evaluative, regulatory, and operational frameworks governing other DTx and medical devices. Such a transition is vital for moving immersive technologies from adjunctive or experimental status to integral components of evidence-based clinical care, enhancing patient outcomes and expanding therapeutic options in radiology and radiotherapy [46,47].

### 4.3. Market Growth and Emerging Opportunities for Immersive Digital Therapeutics in Pain and Anxiety Management

The global digital therapeutics (DTx) market is experiencing sustained growth, driven by rising chronic health conditions, demand for non-pharmacological and patient-centered treatments, and growing acceptance of digital health innovations. Chronic conditions, such as persistent pain, anxiety, depression, and other mental health challenges, are major drivers, as traditional pharmaceuticals often present side effects, addiction risks, or incomplete symptom control. According to Roots Analysis, the global DTx market is expected to grow from USD 2.83 billion in 2024 to USD 19.76 billion by 2035, with a CAGR of 19.32% [48].

Technological advancements in artificial intelligence, cloud computing, wearable sensors, and immersive media have enhanced the sophistication and personalization of DTx interventions. Increased regulatory clarity and reimbursement frameworks are facilitating adoption. Immersive technologies, such as virtual reality (VR), augmented reality (AR), and mixed reality (MR), now play a transformative role, offering multisensory, interactive experiences beyond traditional screen-based tools, improving patient engagement, adherence, and outcomes.

These technologies are used in protocols for chronic and acute pain, anxiety, and stress disorders, providing distraction therapy, cognitive-behavioral therapy (CBT), guided relaxation, and biofeedback. VR-based DTx [49] can simulate calming environments or mindfulness sessions, reducing anxiety before and during procedures and improving outcomes.

In the U.S., the DTx market is expanding rapidly, supported by strong healthcare infrastructure and investment. Grand View Research estimates growth from USD 1.8 billion in 2022 to USD 10.5 billion by 2030, with a 25.4% CAGR [50]. The integration of immersive technologies is expected to further accelerate growth as regulatory approvals and clinical validation expand their use beyond chronic pain to procedural, acute, and post-operative contexts.

These projections highlight the recognition of immersive DTx as effective, scalable tools for managing pain, anxiety, and related conditions. Realizing their potential will require continued innovation, robust clinical research, and integration into health systems through standardized protocols, clinician training, and reimbursement strategies.

### 4.4. Complementing Traditional Psychological Therapies with Immersive Technologies: Future Perspectives

The incorporation of immersive technologies—such as virtual reality (VR), augmented reality (AR), and mixed reality (MR)—as adjunctive tools to established psychological treatments like mindfulness, guided imagery, and cognitive-behavioral therapy (CBT) represents a rapidly evolving and highly promising frontier within the digital therapeutics (DTx) landscape. Rather than aiming to replace traditional psychotherapeutic approaches, these immersive tools are designed to complement and enhance them by adding new layers of multisensory engagement, emotional support, and patient empowerment. Through immersive environments, patients can experience therapeutic content in a more vivid, interactive, and embodied manner, which can deepen emotional regulation, increase motivation, and facilitate cognitive restructuring. This multimodal approach allows for therapeutic experiences that extend beyond conventional clinical settings—enabling remote, flexible, and personalized interventions that can be tailored to individual needs and contexts. This evolving integration raises several key areas that are critical for the development, validation, and implementation of immersive psychological interventions within the DTx ecosystem. These key areas represent priorities for research, clinical practice, policy, and industry collaboration.

*Development of Standardized Guidelines*

A foundational step toward broad clinical adoption involves the creation of clear, internationally harmonized guidelines specifically tailored to immersive psychological interventions. These guidelines must ensure the interventions are delivered safely, ethically, and effectively. Important considerations include precise definition of therapeutic goals, aligned to specific clinical populations and psychological conditions, ensuring interventions are targeted and relevant; safety considerations unique to immersive environments, such as the risk of cybersickness (motion sickness induced by VR), emotional overload, or exacerbation of certain psychiatric symptoms, requiring screening and monitoring protocols; standardized intervention protocols, including the type, duration, frequency, and progression of immersive sessions, to enable consistency across clinical settings and research studies; unified outcome measures and assessment tools that capture emotional, cognitive, and physiological responses, facilitating comparison across trials and real-world applications. Organizations such as the Digital Therapeutics Alliance (DTA) have begun to provide classification frameworks and regulatory principles that serve as a strong foundation for such guideline development, encouraging collaboration among stakeholders worldwide [51].

*Regulatory Frameworks and Clinical Validation*

While immersive tools show clear clinical promise, their role as adjuncts to existing therapies presents unique challenges for regulatory approval pathways. Unlike standalone drugs or medical devices, immersive interventions often must be evaluated within the context of complex, combined treatment regimens. Key regulatory challenges include fragmented regulatory standards and pathways across different jurisdictions, which slow harmonized approval and market access; the critical need for large-scale, multi-site randomized controlled trials that rigorously demonstrate the additive or synergistic benefits of immersive adjuncts over standard care alone; the importance of validating safety and tolerability profiles across diverse patient populations, including vulnerable groups, such as children, elderly patients, or those with neuropsychiatric comorbidities. Recent reports from international regulatory bodies emphasize the urgency of modernizing frameworks and fostering global collaboration to establish consistent evaluation criteria. This is particularly relevant within regions such as the European Union, where regulatory pathways are evolving to accommodate innovative digital health technologies [52].

*Reimbursement Pathways*

A critical barrier to widespread clinical adoption is the establishment of sustainable reimbursement frameworks for immersive psychological interventions. As complementary therapies, immersive tools often face greater scrutiny concerning their cost-effectiveness and demonstrable added clinical value. Without reimbursement pathways, scaling beyond pilot projects and early adopters remains challenging. The International Bar Association (IBA) has highlighted reimbursement challenges within the EU and advocates for strategic reforms to recognize digital therapeutics, including immersive tools, as integral components of comprehensive patient care, develop policies that facilitate equitable patient access and incentivize healthcare providers and institutions to adopt these innovative modalities, and encourage value-based pricing models that reflect both clinical benefits and patient-centered outcomes [53].

*Research and Evidence Generation*

Robust, high-quality evidence is the cornerstone of clinical acceptance and guideline development. Emerging research increasingly supports the therapeutic potential of immersive experiences combined with psychological techniques, showing they can enhance emotional regulation and reduce anxiety, distress, and pain in both procedural and chronic illness contexts, improve patient engagement and adherence by creating immersive, interactive therapeutic environments that are more motivating and less burdensome than traditional approaches, and support sustained psychological benefits when used as adjuncts, potentially leading to longer-term improvements in mental health and quality of life. However, a recent comprehensive systematic review underscores the critical need for rigorous clinical validation and real-world effectiveness studies to better understand mechanisms, optimize protocols, and confirm broad applicability [54]. Cutting-edge preprint research exploring VR-based mindfulness interventions further demonstrates promising results in mitigating psychological distress, offering a solid scientific basis for continued innovation and clinical translation [55]. Overall, the path toward full integration of immersive technologies as complementary psychological tools necessitates coordinated, multi-domain efforts. These include aligned research agendas, harmonized regulatory policies, reimbursement strategies that reward value, and clinical training programs that ensure competent and confident use. As the evidence base grows and implementation frameworks mature, immersive DTx modalities are poised to become powerful adjuncts that enrich patient care pathways—enhancing clinical outcomes, improving patient experiences, and expanding access to innovative, personalized psychological therapies. By embracing immersive tools not as replacements for but rather as multi-layered enhancements to established psychological approaches, healthcare systems can offer more holistic, engaging, and effective treatment strategies. This integration is particularly timely in an increasingly digital health landscape where patient expectations, technological capabilities, and clinical needs converge toward more accessible and adaptable care solutions.

### 4.5. Study Limitations

This study employed a mixed methodological approach, combining the comprehensive mapping strengths of a scoping review with the depth and contextualization offered by narrative synthesis. This hybrid design allowed us not only to systematically identify and classify a broad range of studies on immersive technologies for pain and anxiety management in radiology and radiotherapy, but also to interpret emerging themes and clinical implications in a nuanced way. By integrating quantitative and qualitative insights, the review offers a rich, multi-dimensional understanding of the field, providing a valuable foundation for future research and clinical practice.

Nonetheless, as this is not a systematic review or meta-analysis, certain typical methodological procedures were not conducted. For example, formal evaluation of publication bias, such as funnel plots or sensitivity analyses, was not performed. These steps, while important for assessing the robustness and validity of effect sizes, fall outside the primary aims of scoping and narrative reviews, which focus more on breadth of coverage and interpretive synthesis rather than quantitative bias assessment.

Furthermore, the inclusion criteria limited studies to those published in English, which may have excluded relevant research in other languages and thus slightly reduced the global scope of the review. Future research could benefit from including non-English sources to enhance comprehensiveness and cultural relevance.

Overall, these methodological choices reflect the deliberate scope and objectives of the mixed review approach, balancing comprehensive evidence mapping with insightful narrative interpretation, and paving the way for subsequent focused systematic investigations.

## 5. Conclusions

Immersive technologies, such as virtual reality (VR), augmented reality (AR), and mixed reality (MR), are gaining recognition as valuable tools to address pain, anxiety, and distress in radiology and radiotherapy settings. When developed with clear therapeutic protocols and supported by clinical evidence, these technologies move beyond simple distraction to become effective digital therapeutics. Despite promising results, many applications remain in early or exploratory phases, particularly in radiology, highlighting the need for further research, standardized guidelines, and clearer regulatory frameworks. The growing market demand for non-pharmacological interventions and early regulatory approval signal increasing clinical adoption, especially as immersive tools complement established psychological therapies. Continued multidisciplinary collaboration, evidence generation, and policy development will be essential to support the wider integration of immersive digital therapeutics, ultimately enhancing patient care and outcomes.

Building on this foundation, immersive digital therapeutics are well-positioned to transform the landscape of pain, anxiety, and distress management in diagnostic and oncologic care. When immersive interventions are developed with robust therapeutic frameworks and validated through rigorous clinical studies, they become more than mere adjunctive distractions—they evolve into integral components of patient-centered treatment pathways. This transition marks an important milestone in digital health, where technology is harnessed not only for engagement but also for measurable clinical benefit.

Nevertheless, the field remains at a pivotal stage. Many applications are still exploratory, particularly in radiology, and face challenges related to heterogeneity in study designs, patient populations, and intervention protocols. To fully realize their potential, immersive digital therapeutics require clear, internationally harmonized guidelines that address safety, efficacy, and delivery standards tailored to immersive environments. Likewise, regulatory frameworks must evolve to accommodate these hybrid, adjunctive tools, enabling consistent evaluation and smoother approval processes. Equally critical is the establishment of sustainable reimbursement models that recognize the value added by immersive therapies and encourage their adoption in routine care.

The rapid expansion of the immersive digital therapeutics market reflects both the unmet need for non-pharmacological treatment options and the growing confidence of clinicians, patients, and regulators in these technologies. By enhancing traditional psychological therapies like cognitive-behavioral therapy and mindfulness with immersive sensory experiences, these tools offer enriched, multimodal treatment environments that can improve patient engagement, adherence, and outcomes.

Overall, immersive digital therapeutics represent a promising frontier in radiology and radiotherapy capable of enhancing patient wellbeing and clinical outcomes through innovative, evidence-based approaches. Their continued success depends on coordinated efforts spanning research, clinical practice, regulation, and health policy. With sustained investment and collaboration, immersive technologies are poised to become a transformative element of comprehensive patient care, expanding access to effective, personalized, and engaging therapeutic options.

## Figures and Tables

**Table 1:** Summary of main findings from included studies.

First Author and Year	Type of the Study	Comparative	Sample Size	Type of Technology Used	Context	Procedure	Methods/Endpoints	Key Results	Conclusion
Schaake et al., 2024 [27]	Pilot, prospective, randomized controlled trial	Yes (59 VR group vs. 48 SOC)	107	VR	Radiology	PICC placement and thyroid FNA	Pain and anxiety (VAS scale)	Pain reduced by −1.74 (*p* = 0.018), anxiety by −1.60 (*p* = 0.053)	VR reduces pain and anxiety without prolonging procedures
Gullo et al., 2023 [28]	Randomized controlled trial	Yes (50 VR vs. 50 SOC)	100	VR	Radiology	Endovascular interventions	Pain (VAS scale) and anxiety (STAI scale)	Anxiety responders: 76% VR vs. 46% SOC (*p* = 0.004); no significant difference in affective emotional pain (pre–post)	VR self-hypnosis has the potential to improve the management of patients’ distress during radiological procedures
Gao et al., 2022 [29]	Pilot study	Yes (30 VR vs. 30 SOC)	60	VR	Radiotherapy	Chest RT	Questionnaire on RT comprehension, anxiety-related scales, and objective physiological data reflecting BP and respiration of patient	Anxiety scores of the VR group decreased significantly compared with those of the SOC group and there was a significant decrease in systolic BP (*p* < 0.05) and increase in cognitive score (*p* < 0.05)	VR improves understanding of RT and reduces anxiety
Grilo et al., 2023 [30]	Systematic review	No	8 articles included	VR	Radiotherapy	Various anatomical sites	Review of articles regarding educational sessions and similar interventions to prepare patients for treatment, with VR as a learning tool	Anxiety levels decreased with VR educational sessions and throughout the treatment in almost all the studies, although with less homogeneous results	VR methods can enhance cancer patients’ preparation for RT by increasing their understanding of treatment and reducing anxiety
Hernandez et al., 2025 [31]	Pilot study	No	11	VR	Radiotherapy	Lung cancer undergoing concurrent chemotherapy	Questionnaire pre- and post-VR exposure reporting anxiety, pain, and fatigue	Significant improvements were observed in anxiety (*p* = 0.04), fatigue (*p* = 0.03), and pain for preliminary efficacy	VR is safe and potentially effective for distress
Jimenez et al., 2018 [32]	Quasi-experimental study	Yes (19 VR vs. 18 in SOC)	37	VR	Radiotherapy	Breast RT	Repeated surveys on RT knowledge, experience, anxiety	Anxiety level was similar between both groups	VR had no measurable effect on anxiety in this cohort
Bay et al., 2024 [33]	Prospective study	No	50	VR	Radiology	Mammography	Questionnaire including VAS scale	No significant correlation between the perceived pain experienced during mammography and the utilization of VR technology	VR does not reduce pain; perception varies with age
Grange et al., 2024 [34]	Prospective study	No	91	VR	Radiology	Interventional radiology	Questionnaire to evaluate patients’ self-evaluation of pain and anxiety	The mean pain level was 2.5 ± 2.7 before the procedure, 3.3 ± 2.5 during the procedure, and 1.6 ± 2.7 after the procedure. Mean anxiety scores were 4.6 ± 2.9 before the procedure, 3.1 ± 2.7 during the procedure, and 1.1 ± 1.9 after the procedure	VR is feasible, safe, and effective in IR and can be beneficial for pain and anxiety management
Gonzalez et al., 2024 [35]	Prospective study	No	19	VR	Radiotherapy	MRI-guided radiotherapy for abdominal targets	Questionnaire to evaluate patients’ self-evaluation of anxiety	Patients reported the app was “extremely helpful” (28%) or “very helpful” (50%) for reducing anxiety	VR app is effective for patient education and anxiety
Wong et al., 2023 [36]	Prospective, pilot study	No	23	VR	Radiology	Transperineal biopsy/gold seed implantation in prostate cancer patients	Questionnaire including VAS scale for pain and distress	83% of participants with pain scores above the mean and 80% with anxiety scores above the mean agreed that they would be willing to try VR	VR method is useful for patients with high pain/distress
Cornelis et al., 2019 [37]	Review	NA	NA	VR	Radiology	Interventional radiology	Review of articles regarding the techniques of sedation in interventional radiology	None of these included studies in the review concerned directly the use of VR as digital sedation in IR	Despite the limited evidence, digital sedation supported by VR seemed to be a promising approach for interventional radiology
Varnier et al., 2021 [38]	Pilot comparative study	Yes (14 patients in VR group vs. 21 in SOC)	35	VR	Radiotherapy	Uterovaginal brachytherapy applicators’ removal	Pain and anxiety (VAS scale)	The mean VAS-anxiety was 2.9 before and 2.7 at the peak in the VR group versus 4.1 and 1.6, respectively, in the control group. The mean VAS-pain was 1.0 before, 3.1 at the peak, and 0.4 after the procedure in the experimental group, versus 1.8, 2.0, and 0.6, respectively, in the control group	VR is a feasible alternative to gas sedation
Alanazi et al., 2022 [39]	Review	NA	NA	VR	Radiotherapy	Pediatric treatment	Review of articles to determine the potential benefits of VR technology while delivering radiotherapy to pediatric patients	Before the start of radiotherapy, pediatric patients have the opportunity to get used to the VR environment in a safe room. This helps to reduce anxiety and ease the transition to treatment without creating a sense of dislocation	VR has the potential to reduce anxiety in pediatrics during treatment pathway
Perenic et al., 2023 [40]	Prospective study	Yes (75 VR vs. 78 in SOC)	153	VR	Radiology	Transrectal MRI-guided prostate biopsy	Pain (VAS scale)	The mean pain score at day zero was, respectively, 3.4 (±2.5) and 2.9 (±2.3) for SOC and VR (*p =* 0.203). The mean pain score at day zero was significantly lower in naive patients with VR (2.7 (±2.0)) than in naive patients with SOC (3.8 (±2.5), *p* = 0.012)	VR reduces pain in biopsy-naive patients

Output of the scoping review. BP: blood pressure; FNA: fine-needle aspiration; IR: interventional radiology; MRI: magnetic resonance imaging; NA: not applicable; PICC: peripherally inserted central catheter; RT: radiotherapy; SOC: standard of care; STAI: State-Trait Anxiety Inventory; VAS: visual analog scale; VR: virtual reality.

## Data Availability

No new data were produced with the review.

## Supplementary Materials

The following supporting information can be downloaded at: https://www.mdpi.com/article/10.3390/diagnostics15172174/s1, Table S1: Search keys. Table S2: Preferred Reporting Items for Systematic Reviews and Meta-Analyses extension for Scoping Reviews (PRISMA-ScR) Checklist; S2: Sketch of the Overviewed Studies.

## References

[1] Wang J., Doan L.V. (2024). Clinical pain management: Current practice and recent innovations in research. Cell Rep. Med..

[2] Walker J., van Niekerk M., Hobbs H., Toynbee M., Magill N., Bold R., Hampsey E., Harriss E., Frost C., Sharpe M. (2021). The prevalence of anxiety in general hospital inpatients: A systematic review and meta-analysis. Gen. Hosp. Psychiatry.

[3] Lederer A.-K., Manteufel I., Knott A., Müller A., Kousoulas L., Werthmann P.G., Klein A.C., Huber R. (2024). The Impact of Surgery-Related Emotional Distress on Long-Term Outcome After Colorectal Surgery: An Observational Follow-Up. J. Clin. Med..

[4] Morone N.E., Belnap B.H., He F., Mazumdar S., Weiner D.K., Rollman B.L. (2013). Pain adversely affects outcomes to a collaborative care intervention for anxiety in primary care. J. Gen. Intern. Med..

[5] Rhudy J.L., Meagher M.W. (2001). The role of emotion in pain modulation. Curr. Opin. Psychiatry.

[6] Kessler R.C., Sonnega A., Bromet E., Hughes M., Nelson C.B. (1995). Posttraumatic stress disorder in the National Comorbidity Survey. Arch. Gen. Psychiatry.

[7] Hyland S.J., Brockhaus K.K., Vincent W.R., Spence N.Z., Lucki M.M., Howkins M.J., Cleary R.K. (2021). Perioperative Pain Management and Opioid Stewardship: A Practical Guide. Healthcare.

[8] Meléndez J.C., McCrank E. (1993). Anxiety-related reactions associated with magnetic resonance imaging examinations. JAMA.

[9] Katz R.C., Wilson L., Frazer N. (1994). Anxiety and its determinants in patients undergoing magnetic resonance imaging. J. Behav. Ther. Exp. Psychiatry.

[10] Delewi R., Vlastra W., Rohling W.J., Wagenaar T.C., Zwemstra M., Meesterman M.G., Vis M.M., Wykrzykowska J.J., Koch K.T., de Winter R.J. (2017). Anxiety levels of patients undergoing coronary procedures in the catheterization laboratory. Int. J. Cardiol..

[11] Fabian A., Rühle A., Domschikowski J., Trommer M., Wegen S., Becker J.N., Wurschi G., Boeke S., Sonnhoff M., Fink C.A. (2023). Psychosocial distress in cancer patients undergoing radiotherapy: A prospective national cohort of 1042 patients in Germany. J. Cancer Res. Clin. Oncol..

[12] Andersen B.L., Karlsson J.A., Anderson B., Tewfik H.H. (1984). Anxiety and cancer treatment: Response to stressful radiotherapy. Health Psychol..

[13] Garland E.L., Froeliger B., Howard M.O. (2014). Mindfulness training targets neurocognitive mechanisms of addiction at the attention-appraisal-emotion interface. Front. Psychiatry.

[14] Zeidan F., Grant J.A., Brown C.A., McHaffie J.G., Coghill R.C. (2012). Mindfulness meditation-related pain relief: Evidence for unique brain mechanisms in the regulation of pain. Neurosci. Lett..

[15] https://www.physio-pedia.com/Psychological_Approaches_to_Pain_Management.

[16] Toussaint L., Nguyen Q.A., Roettger C., Dixon K., Offenbächer M., Kohls N., Hirsch J., Sirois F. (2021). Effectiveness of Progressive Muscle Relaxation, Deep Breathing, and Guided Imagery in Promoting Psychological and Physiological States of Relaxation. Evid. Based Complement. Alternat. Med..

[17] Lee H.J. (2021). Digital therapeutics in pain medicine. Korean J. Pain.

[18] Lastrucci A., Votta C., Serventi E., Cornacchione P., Francioni S., Wandael Y., Talamonti C., Ricci R. (2024). The application of virtual environment radiotherapy for RTT training: A scoping review. J. Med. Imaging Radiat. Sci..

[19] Lastrucci A., Wandael Y., Barra A., Ricci R., Maccioni G., Pirrera A., Giansanti D. (2024). Exploring Augmented Reality Integration in Diagnostic Imaging: Myth or Reality?. Diagnostics.

[20] Teh J.J., Pascoe D.J., Hafeji S., Parchure R., Koczoski A., Rimmer M.P., Khan K.S., Al Wattar B.H. (2024). Efficacy of virtual reality for pain relief in medical procedures: A systematic review and meta-analysis. BMC Med..

[21] Janecký D., Kučera E., Haffner O., Košutzká Z., Martiš P. (2025). The Use of Mixed Reality for Exposure Therapy to Improve Phobia Handling. Int. J. Cogn. Behav. Ther..

[22] Mittal A., Wakim J., Huq S., Wynn T. (2024). Effectiveness of XR Reality in Reducing Perceived Pain and Anxiety Among Patients Within a Hospital System: Protocol for a Mixed Methods Study. JMIR Res. Protoc..

[23] Burridge N., Sillence A., Teape L., Clark B., Bruce E., Armoogum J., Leloch D., Spathis A., Etkind S. (2025). Virtual reality reduces anxiety and pain in acute hospital palliative care: Service evaluation. BMJ Support. Palliat. Care.

[24] Tricco A.C., Lillie E., Zarin W., O’Brien K.K., Colquhoun H., Levac D., Moher D., Peters M.D.J., Horsley T., Weeks L. (2018). PRISMA extension for scoping reviews (PRISMA-ScR): Checklist and explanation. Ann. Intern. Med..

[25] Arksey H., O’Malley L. (2005). Scoping studies: Towards a methodological framework. Int. J. Soc. Res. Methodol. Theory Pract..

[26] Kapoor M.C. (2016). Types of studies and research design. Indian J. Anaesth..

[27] Schaake R., Leopold I., Sandberg A., Zenk B., Shafer L., Yu D., Lu X., Theingi S., Udongwo A., Cohen G.S. (2024). Virtual Reality for the Management of Pain and Anxiety for IR Procedures: A Prospective, Randomized, Pilot Study on Digital Sedation. J. Vasc. Interv. Radiol..

[28] Gullo G., Rotzinger D.C., Colin A., Frossard P., Gudmundsson L., Jouannic A.M., Qanadli S.D. (2023). Virtually Augmented Self-Hypnosis in Peripheral Vascular Intervention: A Randomized Controlled Trial. Cardiovasc. Intervent. Radiol..

[29] Gao J., Liu S., Zhang S., Wang Y., Liang Z., Feng Q., Hu M., Zhang Q. (2022). Pilot Study of a Virtual Reality Educational Intervention for Radiotherapy Patients Prior to Initiating Treatment. J. Cancer Educ..

[30] Grilo A.M., Almeida B., Rodrigues C., Isabel Gomes A., Caetano M. (2023). Using virtual reality to prepare patients for radiotherapy: A systematic review of interventional studies with educational sessions. Tech. Innov. Patient Support. Radiat. Oncol..

[31] Hernandez R., Nisar H., Kesavadas T.K., McGee M.C., Gerstner G.J., Martinez A., Boyce C., Ashrafi S.A., Addington E.L., Matthews A.K. (2025). Assessing safety and feasibility of virtual reality intervention in patients with lung cancer: A pilot study. Support. Care Cancer.

[32] Jimenez Y.A., Cumming S., Wang W., Stuart K., Thwaites D.I., Lewis S.J. (2018). Patient education using virtual reality increases knowledge and positive experience for breast cancer patients undergoing radiation therapy. Support. Care Cancer.

[33] Bay B.N., Voyvoda N., Arifoğlu M. (2024). An initial investigation into the use of virtual reality (VR) glasses on self-reported pain perception during mammography. Radiography.

[34] Grange L., Grange R., Bertholon S., Morisson S., Martin I., Boutet C., Grange S. (2024). Virtual reality for interventional radiology patients: A preliminary study. Support. Care Cancer.

[35] Gonzalez B.D., Choo S., Janssen J.J., Hazelton J., Latifi K., Leach C.R., Bailey S., Jim H.S.L., Oswald L.B., Woolverton M. (2024). Novel Virtual Reality App for Training Patients on MRI-guided Radiation Therapy. Adv. Radiat. Oncol..

[36] Wong J., McGuffin M., Smith M., Loblaw D.A. (2023). The use of virtual reality hypnosis for prostate cancer patients during transperineal biopsy/gold seed implantation: A needs assessment study. J. Med. Imaging Radiat. Sci..

[37] Cornelis F.H., Monard E., Moulin M.A., Vignaud E., Laveissiere F., Ben Ammar M., Nouri-Neuville M., Barral M., Lombart B. (2019). Sedation and analgesia in interventional radiology: Where do we stand, where are we heading and why does it matter?. Diagn. Interv. Imaging.

[38] Varnier R., Brière O., Brouillard T., Martel-Lafay I., Serre A.A., Couillet A., Chvetzoff G., Freulet C., Pommier P. (2021). Virtual reality distraction during uterovaginal brachytherapy applicators’ removal: A pilot comparative study. Brachytherapy.

[39] Alanazi A., Ashour F., Aldosari H., Aldosari B. (2022). The Impact of Virtual Reality in Enhancing the Quality of Life of Pediatric Oncology Patients. Stud. Health Technol. Inform..

[40] Perenic E., Grember E., Bassard S., Koutlidis N. (2023). Impact of virtual reality on pain management in transrectal MRI-guided prostate biopsy. Front. Pain Res..

[41] Digital Therapeutics Alliance Understanding DTx. https://dtxalliance.org/understanding-dtx/.

[42] Maddox T., Oldstone L., Sparks C.Y., Sackman J., Oyao A., Garcia L., Maddox R.U., Ffrench K., Garcia H., Adair T. (2023). In-Home Virtual Reality Program for Chronic Lower Back Pain: A Randomized Sham-Controlled Effectiveness Trial in a Clinically Severe and Diverse Sample. Mayo Clin. Proc. Digit. Health.

[43] https://www.relievrx.com/.

[44] Digital Therapeutics Alliance Guidance to Industry: Classification of Digital Health Technologies. https://dtxalliance.org/wp-content/uploads/2023/06/Guidance-to-Industry-Classification-of-Digital-Health-Technologies-2023Jun05.pdf.

[45] Lougheed T. (2019). How “digital therapeutics” differ from traditional health and wellness apps. CMAJ.

[46] WHO Recommendations on Digital Interventions for Health System Strengthening. https://www.who.int/publications/i/item/9789241550505.

[47] NICE NICE Recommends 8 Digitally Enabled Therapies to Treat Depression and Anxiety. https://www.nice.org.uk/news/articles/nice-recommends-8-digitally-enabled-therapies-to-treat-depression-and-anxiety.

[48] https://www.rootsanalysis.com/reports/digital-therapeutics-market/208.html.

[49] https://www.marknteladvisors.com/research-library/digital-therapeutics-market.html.

[50] https://www.grandviewresearch.com/industry-analysis/us-digital-therapeutics-market.

[51] https://dtxalliance.org/wp-content/uploads/2023/06/DTA_FS_DHT-Ecosystem-Categorization.pdf.

[52] https://www.ey.com/en_ie/insights/life-sciences/navigating-the-digital-wave-dtx-regulations-in-europe-and-beyond.

[53] https://www.ibanet.org/patient-access-new-digital-therapies-eu?sap-outbound-id=7FDD82BC482159FEA6728E55417978A58710C037.

[54] Gomis-Pastor M., Berdún J., Borrás-Santos A., De Dios López A., Fernández-Montells Rama B., García-Esquirol Ó., Gratacòs M., Ontiveros Rodríguez G.D., Pelegrín Cruz R., Real J. (2024). Clinical Validation of Digital Healthcare Solutions: State of the Art, Challenges and Opportunities. Healthcare.

[55] Yildirim C., O’Grady T. (2021). The efficacy of a virtual reality-based mindfulness intervention. arXiv.

