# Supplementary material for: A Mixed Scoping and Narrative Review of Immersive Technologies Applied to Patients for Pain, Anxiety, and Distress in Radiology and Radiotherapy
Source: Diagnostics (Basel). 2025 Aug 27;15(17):2174. doi: 10.3390/diagnostics15172174 (PMC12428699; doi:10.3390/diagnostics15172174)
Supplement: Supplementary file 1 [file diagnostics-15-02174-s001.zip › diagnostics-3819189-supplementary.pdf]

diagnostics

MDPI

Review

A *Mixed Scoping and Narrative Review* of Immersive Technologies Applied to Patients for Pain, Anxiety, and Distress in Radiology and Radiotherapy

Andrea Lastrucci [1], Nicola Iosca [1], Giorgio Busto [2], Yannick Wandael [1], Angelo Barra [1], Mirko Rossi [3], Ilaria Morelli [4], Antonia Pirrera [3], Isacco Desideri [5], Renzo Ricci [1], Lorenzo Livi [5] and Daniele Giansanti [3,*]

1 Department of Allied Health Professions, Azienda Ospedaliero-Universitaria Careggi, 50134 Florence, Italy
2 Neuroradiology Unit, Department of Radiology, Careggi University Hospital, Florence, Italy
3 Centro TISP, Istituto Superiore di Sanità, via Regina Elena 299, 00161 Rome, Italy
4 Medical Oncology, Santa Maria delle Croci hospital, Ravenna AUSL Romagna Italy
5 Department of Experimental and Clinical Biomedical Sciences "M. Serio", University of Florence, 50134 Florence, Italy
* Correspondence: Daniele.giansanti@iss.it

SUPPLEMENTARY MATERIAL

S1 Complementary Tables

In this section, we report Table S1 with the search keys and Table S2 with the scoping review checklist.

Table S1. Search keys.

Database	Search String
PubMed	(("pain"[Title/Abstract] OR "discomfort"[Title/Abstract] OR "Pain"[MeSH Terms]) AND ("virtual reality"[Title/Abstract] OR "augmented reality"[Title/Abstract] OR "extended reality"[Title/Abstract] OR "mixed reality"[Title/Abstract] OR "XR"[Title/Abstract] OR "Virtual Reality"[MeSH Terms]) AND ("radiotherapy"[Title/Abstract] OR "radiation therapy"[Title/Abstract] OR "radiology"[Title/Abstract] OR "Radiotherapy"[MeSH Terms] OR "Radiology"[MeSH Terms]))
Embase	("pain"/exp OR pain:ti,ab OR discomfort:ti,ab) AND ("virtual reality"/exp OR "augmented reality":ti,ab OR "extended reality":ti,ab OR "mixed reality":ti,ab OR xr:ti,ab OR "virtual reality":ti,ab) AND ("radiotherapy"/exp OR "radiation therapy":ti,ab OR radiology:ti,ab)
Scopus	TITLE-ABS-KEY ("pain" OR "discomfort") AND TITLE-ABS-KEY ("virtual reality" OR "augmented reality" OR "extended reality" OR "mixed reality" OR "xr") AND TITLE-ABS-KEY ("radiotherapy" OR "radiation therapy" OR "radiology")
WoS	TS = ("pain" OR "discomfort") AND TS = ("virtual reality" OR "augmented reality" OR "extended reality" OR "mixed reality" OR XR) AND TS = ("radiotherapy" OR "radiation therapy" OR "radiology")

Table S2. Preferred Reporting Items for Systematic Reviews and Meta-Analyses extension for Scoping Reviews (PRISMA-ScR) Checklist.

SECTION	ITEM	PRISMA-ScR CHECKLIST ITEM	REPORTED ON PAGE #
**TITLE**			
Title	1	Identify the report as a scoping review.	1
**ABSTRACT**			
Structured summary	2	Provide a structured summary that includes (as applicable) background, objectives, eligibility criteria, sources of evidence, charting methods, results, and conclusions that relate to the review questions and objectives.	1–2
**INTRODUCTION**			
Rationale	3	Describe the rationale for the review in the context of what is already known. Explain why the review questions/objectives lend themselves to a scoping review approach.	3
Objectives	4	Provide an explicit statement of the questions and objectives being addressed with reference to their key elements (e.g., population or participants, concepts, and context) or other relevant key elements used to conceptualize the review questions and/or objectives.	3–4
**METHODS**			
Protocol and registration	5	Indicate whether a review protocol exists, state if and where it can be accessed (e.g., a Web address), and, if available, provide registration information, including the registration number.	NA
Eligibility criteria	6	Specify characteristics of the sources of evidence used as eligibility criteria (e.g., years considered, language, and publication status) and provide a rationale.	5–6
Information sources*	7	Describe all information sources in the search (e.g., databases with dates of coverage and contact with authors to identify additional sources), as well as the date the most recent search was executed.	5
Search	8	Present the full electronic search strategy for at least 1 database, including any limits used, such that it could be repeated.	Supplementary material
Selection of sources of evidence†	9	State the process for selecting sources of evidence (i.e., screening and eligibility) included in the scoping review.	6
Data charting process‡	10	Describe the methods of charting data from the included sources of evidence (e.g., calibrated forms or forms that have been tested by the team before their use, and whether data charting was done independently or in duplicate) and any processes for obtaining and confirming data from investigators.	6–7
Data items	11	List and define all variables for which data were sought and any assumptions and simplifications made.	6–7
Critical appraisal of individual sources of evidence§	12	If done, provide a rationale for conducting a critical appraisal of included sources of evidence, and describe the methods used and how this information was used in any data synthesis (if appropriate).	NA
Synthesis of results	13	Describe the methods of handling and summarizing the data that were charted.	7
**RESULTS**			
Selection of sources of evidence	14	Give numbers of sources of evidence screened, assessed for eligibility, and included in the review, with reasons for exclusions at each stage, ideally using a flow diagram.	10
Characteristics of sources of evidence	15	For each source of evidence, present characteristics for which data were charted and provide the citations.	15–19

SECTION	ITEM	PRISMA-ScR CHECKLIST ITEM	REPORTED ON PAGE #
Critical appraisal within sources of evidence	16	If done, present data on critical appraisal of included sources of evidence (see item 12).	NA
Results of individual sources of evidence	17	For each included source of evidence, present the relevant data that were charted that relate to the review questions and objectives.	11–14
Synthesis of results	18	Summarize and/or present the charting results as they relate to the review questions and objectives.	11–14
**DISCUSSION**			
Summary of evidence	19	Summarize the main results (including an overview of concepts, themes, and types of evidence available), link to the review questions and objectives, and consider the relevance to key groups.	20-23
Limitations	20	Discuss the limitations of the scoping review process.	26
Conclusions	21	Provide a general interpretation of the results with respect to the review questions and objectives, as well as potential implications and/or next steps.	26-27
**FUNDING**			
Funding	22	Describe sources of funding for the included sources of evidence, as well as sources of funding for the scoping review. Describe the role of the funders of the scoping review.	27

JBI = Joanna Briggs Institute; PRISMA-ScR = Preferred Reporting Items for Systematic Reviews and Meta-Analyses extension for Scoping Reviews. * Where sources of evidence (see second footnote) are compiled from, such as bibliographic databases, social media platforms, and Web sites. † A more inclusive/heterogeneous term used to account for the different types of evidence or data sources (e.g., quantitative and/or qualitative research, expert opinion, and policy documents) that may be eligible in a scoping review, as opposed to only studies. This is not to be confused with information sources (see first footnote). ‡ The frameworks by Arksey and O'Malley (6) and Levac and colleagues (7) and the JBI guidance (4, 5) refer to the process of data extraction in a scoping review as data charting. § The process of systematically examining research evidence to assess its validity, results, and relevance before using it to inform a decision. This term is used for items 12 and 19 instead of "risk of bias" (which is more applicable to systematic reviews of interventions) to include and acknowledge the various sources of evidence that may be used in a scoping review (e.g., quantitative and/or qualitative research, expert opinion, and policy document). From: Tricco AC, Lillie E, Zarin W, O'Brien KK, Colquhoun H, Levac D, et al. PRISMA Extension for Scoping Reviews (PRISMAScR): Checklist and Explanation. Ann Intern Med. 2018;169:467–473. doi: 10.7326/M18-0850.

S2 Sketch of the Overviewed Studies

The following studies, numbered [27–40], have been systematically identified and are presented here according to a structured analytical framework. This framework aims to provide a clear and comprehensive overview of each study's key features and findings in the context of virtual reality (VR) and immersive technologies applied to pain, anxiety, and distress management in clinical settings.

Each study is summarized using the following categories:

Study Aims and Design: This section outlines the primary objectives of the study and the research design employed, such as randomized controlled trials (RCTs), pilot studies, or observational research. It provides insight into the study's methodological rigor and scope.

Digital/Immersive Technology Employed: Here, the specific VR or immersive technology used in the intervention is described, including the type of hardware (e.g., VR

headsets), software environments, and the nature of the immersive experience (e.g., distraction, self-hypnosis, or guided relaxation). This information highlights the technological approaches leveraged to influence patient outcomes.

Patient Population and Clinical Context: This section identifies the demographic and clinical characteristics of the participants, detailing the medical conditions or procedures involved and the healthcare setting in which the intervention took place. Understanding this context is essential to gauge the applicability and generalizability of the results.

Outcomes Measured and Results: The key clinical or psychological outcomes measured (such as pain, anxiety, procedural tolerance, or quality of life) are summarized along with the main findings. This offers a concise view of the efficacy or impact of the VR intervention.

Contribution to Pain, Anxiety, and Distress Management: Finally, each summary includes an assessment of how the intervention contributes to managing pain, anxiety, or distress, considering its potential clinical benefits, implementation feasibility, and any noted limitations or areas for future research.

This structured approach facilitates a comprehensive yet concise understanding of the current evidence on the use of VR and immersive technologies in supporting patient comfort and psychological wellbeing during medical procedures. It also provides a foundation for identifying gaps in knowledge and informing future innovations in this rapidly evolving field.

Ref. [27]

Study Aims and Design

This prospective, randomized pilot trial evaluated the effectiveness of VR as a digital sedation adjunct during interventional radiology (IR) procedures, specifically PICC placements. Participants were randomized to either VR or standard of care (SOC), with primary outcomes including procedural pain and anxiety.

Digital/Immersive Technology Employed

Participants in the intervention group wore a VR headset delivering immersive content during the procedure. The exact environment was not detailed in the abstract, but the use suggests distraction and sensory immersion to reduce discomfort.

Patient Population and Clinical Context

Adult patients undergoing peripherally inserted central catheter (PICC) placement in an IR setting.

Outcomes Measured and Results

Anxiety was assessed using a visual analogue scale (VAS). There was a clinically meaningful reduction of −1.60 points (SE ± 0.81; $p = 0.053$) favoring the VR group, also with evidence of pain reduction—all achieved without prolonging procedure time

Contribution to Pain, Anxiety, and Distress Management

This pilot demonstrates that VR can act as an effective nonpharmacologic sedation adjunct, reducing both anxiety and pain in IR procedures. Importantly, it achieves this without extending procedural duration, suggesting potential for streamlined integration into clinical workflows. Limitations include small sample size and lack of a sham or control VR.

Ref. [28]

Study Aims and Design

This RCT tested the efficacy of virtually augmented self-hypnosis (VA-HYPO)—a VR-based self-hypnosis technique—compared to treatment as usual during endovascular interventions.

Digital/Immersive Technology Employed

All participants in the intervention arm used a VR headset running the Oncomfort™ "Aqua" module: an immersive underwater scenario combined with guided breathing and self-hypnosis induction designed to elicit a dissociative, relaxed state.

Patient Population and Clinical Context

Adults undergoing peripheral vascular interventions (catheter-based), randomized 1:1 to VA-HYPO or TAU.

Outcomes Measured and Results

Primary measures included procedural pain and anxiety. While precise numerical outcomes are not detailed in the abstract, the authors concluded that VA-HYPO significantly reduced both metrics compared to TAU.

Contribution to Pain, Anxiety, and Distress Management

This study introduces self-hypnotic VR as a patient-driven, immersive nonpharmacologic method. It highlights VA-HYPO's potential to empower patients, reducing dependency on clinician-guided interventions and improving tolerability during minimally invasive vascular procedures.

Ref. [29]

Study Aims and Design

An RCT pilot tested whether a VR-based educational module, delivered before starting radiotherapy, could improve patient comprehension and reduce pre-treatment anxiety. Sixty participants were randomized to VR education or standard care.

Digital/Immersive Technology Employed

Participants in the VR group used a headset to explore a virtual radiotherapy walkthrough—covering procedure steps, positioning, and machine sounds—prior to their first session.

Patient Population and Clinical Context

Adults beginning external beam radiotherapy for various cancers.

Outcomes Measured and Results

Outcomes included knowledge assessment, anxiety (STAI and VAS), and systolic blood pressure. Post-intervention, the VR group had significantly reduced anxiety, improved cognitive comprehension, and lower systolic BP compared to the control.

Contribution to Pain, Anxiety, and Distress Management

This study illustrates VR's role as an educational tool that attenuates uncertainty-driven anxiety. By demystifying RT procedures through simulated exposure, it fosters understanding and calm—even before treatment begins.

Ref [30]

Study Aims and Design

This systematic review analyzed 7 interventional studies (total n = 376) assessing VR-based educational sessions for adult radiotherapy patients.

Digital/Immersive Technology Employed

All reviewed interventions involved VR modules used before or during RT, varying in detail but consistently providing immersive simulation of patient positioning, machine sounds, and procedural steps.

Patient Population and Clinical Context

Adult oncology patients undergoing external beam radiotherapy for diverse tumor types.

Outcomes Measured and Results

Most studies reported significant improvements in patient knowledge and reductions in treatment-related anxiety, though effect sizes varied. Anxiety reduction was consistent but less uniform across studies.

Contribution to Pain, Anxiety, and Distress Management

This review synthesizes evidence reinforcing VR's value as a preparation tool that enhances cognitive readiness and emotional stability. It underlines its acceptability and feasibility across diverse RT settings, while noting heterogeneity in intervention formats and outcomes.

Ref. [31]

Study Aims and Design

A phase-1, single-arm pilot evaluating the safety, usability, and preliminary efficacy of a custom VR program ("Joviality™") targeting emotional wellbeing in lung cancer patients undergoing concurrent chemoradiation.

Digital/Immersive Technology Employed

Participants engaged in a 25-minute 3D VR session aimed at fostering positive emotions, guided by Joviality™ software. Physiological and usability metrics were tracked.

Patient Population and Clinical Context

Eleven patients (mean age ~66), undergoing lung cancer treatment. Exclusion criteria included significant motion sickness or prior VR intolerance.

Outcomes Measured and Results

Primary outcomes: safety (Simulator Sickness Questionnaire), sense of presence, and usability (System Usability Scale). Secondary: anxiety, fatigue, and pain. Results showed no significant motion sickness, high presence (5.26/7), strong usability (78.2), and statistically significant improvements in anxiety ($p = 0.04$), fatigue ($p = 0.03$), and pain.

Contribution to Pain, Anxiety, and Distress Management

This first-in-population study affirms VR's feasibility and acceptability in lung cancer care, with early signals of efficacy in reducing distress, fatigue, and pain. It establishes groundwork for larger phase-2/3 trials, underscoring the potential of emotion-focused VR therapy alongside chemoradiation protocols.

Ref [32]

Study Aims and Design

Pilot mixed-methods study (quantitative and qualitative) evaluating the effectiveness of a VR educational session.

Digital/Immersive Technology Employed

Immersive VR headset simulating the radiotherapy environment and procedures with a guided walkthrough to familiarize patients.

Patient Population and Clinical Context

Forty-three breast cancer patients prior to starting radiotherapy.

Outcomes Measured and Results

Significant increase in patient knowledge about radiotherapy.

Improved subjective patient experience with reduced anticipatory anxiety.

Patients reported a greater sense of control and emotional preparedness.

Contribution to Pain, Anxiety, and Distress Management

VR indirectly reduced anxiety and distress by improving information delivery and patient familiarization, enhancing perceived control. Direct effects on pain were not measured.

Ref. [33]

Full Reference

Bay BN, Voyvoda N, Arifoğlu M. An initial investigation into the use of virtual reality (VR) glasses on self-reported pain perception during mammography. *Radiography (Lond).* 2024 Aug;30(5):1363-1367. doi: 10.1016/j.radi.2024.07.021.

Objectives and Study Design

This pilot randomized study aimed to evaluate whether wearing VR glasses during mammography could reduce self-reported pain perception. Mammography is often

associated with discomfort and anxiety, and this study sought to investigate VR as a distraction technique to alleviate these symptoms.

Immersive/Digital Technology Used

Participants wore VR glasses delivering immersive visual content designed to distract and engage the patient during the mammogram procedure.

Population and Clinical Context

Women undergoing routine mammographic screening at a radiology department, a procedure known for causing discomfort and distress.

Outcomes Measured and Results

Pain perception was assessed using validated self-report scales. The VR group reported statistically significant lower pain scores compared to controls. Participants also reported improved comfort and willingness to undergo future mammograms.

Contribution to Pain, Anxiety, and Distress Management

The study highlights VR's potential as a non-pharmacologic, cost-effective method to reduce pain and anxiety during mammography, potentially improving patient compliance with screening programs.

Ref. [34]

Full Reference

Grange L, Grange R, Bertholon S, Morisson S, Martin I, Boutet C, Grange S. Virtual reality for interventional radiology patients: a preliminary study. *Support Care Cancer*. 2024 Jun 7;32(7):416. doi: 10.1007/s00520-024-08621-0.

Objectives and Study Design

This preliminary prospective study explored the feasibility and effectiveness of VR immersion in reducing anxiety among patients undergoing interventional radiology procedures. The focus was on psychological comfort and patient experience improvement.

Immersive/Digital Technology Used

Patients wore VR headsets that immersed them in calming, interactive virtual environments during radiologic interventions.

Population and Clinical Context

Cancer patients undergoing minimally invasive but anxiety-provoking radiological procedures, which may be stressful due to their invasive nature and clinical implications.

Outcomes Measured and Results

Anxiety was measured pre- and post-intervention using standardized scales. VR significantly reduced anxiety levels, and patients reported a better overall procedural experience with minimal adverse effects.

Contribution to Pain, Anxiety, and Distress Management

VR provided an effective distraction that improved patient wellbeing during procedures, suggesting a role for VR as an adjunct to traditional sedation and analgesia in interventional radiology.

Ref [35]

Objectives and Study Design

This study developed and piloted a novel VR application aimed at educating patients scheduled for MRI-guided radiation therapy. The primary goal was to reduce pre-treatment anxiety by familiarizing patients with the treatment environment and procedure through immersive simulation.

Immersive/Digital Technology Used

An interactive VR app simulating the radiation therapy environment and procedure, enabling patients to virtually experience the therapy setup and process prior to actual treatment.

Population and Clinical Context

Oncology patients undergoing MRI-guided radiation therapy, a complex and potentially anxiety-inducing treatment.

Outcomes Measured and Results

Patients who used the VR app demonstrated significantly reduced anxiety and improved understanding of the procedure compared to controls. Feedback indicated high usability and acceptability.

Contribution to Pain, Anxiety, and Distress Management

This VR training tool supports patient empowerment through education, alleviating fear of the unknown and enhancing psychological readiness for complex cancer treatments.

Ref [36]

Full Reference

Wong J, McGuffin M, Smith M, Loblaw DA. The use of virtual reality hypnosis for prostate cancer patients during transperineal biopsy/gold seed implantation: A needs assessment study. *J Med Imaging Radiat Sci.* 2023 Sep;54(3):429-435. doi: 10.1016/j.jmir.2023.05.004.

Objectives and Study Design

This exploratory needs assessment investigated prostate cancer patients◎interest in and perceived benefits of virtual reality hypnosis (VRH) during invasive procedures, such as transperineal biopsy and gold seed implantation.

Immersive/Digital Technology Used

VR hypnosis combining immersive VR environments with guided hypnotic suggestions aimed at reducing pain and anxiety.

Population and Clinical Context

Men with prostate cancer undergoing invasive diagnostic or treatment procedures associated with discomfort and stress.

Outcomes Measured and Results

Participants reported positive attitudes toward VRH as a complementary method for managing procedural pain and anxiety. Many expressed willingness to try VRH in future procedures.

Contribution to Pain, Anxiety, and Distress Management

The study supports the acceptability of VRH and encourages further trials to validate its efficacy as a non-pharmacologic adjunct for pain and anxiety reduction during urologic interventions.

Ref. [37]

Objectives and Study Design

A narrative review discussing current practices in sedation and analgesia during interventional radiology procedures, addressing challenges and emerging trends, including the potential role of digital and immersive technologies.

Immersive/Digital Technology Used

Discussion of VR and other non-pharmacologic approaches as potential adjuncts or alternatives to traditional sedation.

Population and Clinical Context

Patients undergoing interventional radiology procedures ranging from minimally invasive biopsies to therapeutic interventions.

Outcomes Measured and Results

The review synthesizes evidence on sedation safety, effectiveness, and patient comfort, highlighting a trend toward multimodal approaches incorporating digital distraction techniques.

Contribution to Pain, Anxiety, and Distress Management

Emphasizes the promise of VR and related technologies in reducing sedative drug use,

minimizing side effects, and improving patient experience during radiologic interventions.

Ref. [38]

Objectives and Study Design

Pilot comparative study assessing the effect of VR distraction on pain and anxiety during the removal of brachytherapy applicators in uterovaginal cancer patients.

Immersive/Digital Technology Used

Patients wore VR headsets delivering immersive, calming virtual environments during the removal procedure.

Population and Clinical Context

Women undergoing brachytherapy for gynecologic cancers, specifically during the removal phase known to cause acute pain and distress.

Outcomes Measured and Results

Significant reductions in self-reported pain and anxiety scores were observed in the VR group compared to controls. Patients tolerated the VR intervention well.

Contribution to Pain, Anxiety, and Distress Management

This study provides evidence supporting VR as an effective, non-invasive adjunct to pain and anxiety management in oncological brachytherapy.

Ref [39]

Objectives and Study Design

This exploratory study evaluated the impact of VR interventions on the quality of life in pediatric oncology patients during hospitalization.

Immersive Digital Technology Used

Various VR applications providing games, relaxation exercises, and interactive environments designed to distract and engage children during treatment.

Population and Clinical Context

Children and adolescents undergoing cancer treatment in inpatient settings.

Outcomes Measured and Results

VR use was associated with improved mood, decreased perception of pain, and better overall quality of life indicators. Feedback from patients and caregivers was highly positive.

Contribution to Pain, Anxiety, and Distress Management

VR shows promise as a supportive care tool in pediatric oncology, improving emotional wellbeing and coping during challenging treatments.

Ref [40]

Objectives and Study Design

A randomized controlled trial assessing the efficacy of VR-assisted preoperative education on anxiety, knowledge retention, and postoperative outcomes among breast cancer surgery patients.

Immersive/Digital Technology Used

VR modules providing detailed, interactive preoperative education including virtual walkthroughs of the surgical procedure and postoperative care.

Population and Clinical Context

Women scheduled for breast cancer surgery, facing preoperative anxiety and information gaps.

Outcomes Measured and Results

Patients receiving VR education showed significantly lower preoperative anxiety, better understanding of their surgery, and improved postoperative satisfaction. No adverse effects were reported.

Contribution to Pain, Anxiety, and Distress Management

This study supports VR as a valuable tool for enhancing patient education, reducing anxiety, and potentially improving clinical outcomes through better patient preparedness.
